# Distinct DNA Methylation Patterns of Subependymal Giant Cell Astrocytomas in Tuberous Sclerosis Complex

**DOI:** 10.1007/s10571-021-01157-5

**Published:** 2021-10-28

**Authors:** Anika Bongaarts, Caroline Mijnsbergen, Jasper J. Anink, Floor E. Jansen, Wim G. M. Spliet, Wilfred F. A. den Dunnen, Roland Coras, Ingmar Blümcke, Werner Paulus, Victoria E. Gruber, Theresa Scholl, Johannes A. Hainfellner, Martha Feucht, Katarzyna Kotulska, Sergiusz Jozwiak, Wieslawa Grajkowska, Anna Maria Buccoliero, Chiara Caporalini, Flavio Giordano, Lorenzo Genitori, Figen Söylemezoğlu, José Pimentel, David T. W. Jones, Brendon P. Scicluna, Antoinette Y. N. Schouten-van Meeteren, Angelika Mühlebner, James D. Mills, Eleonora Aronica

**Affiliations:** 1 Department of Neuro Pathology, Amsterdam UMC, Location AMC, University of Amsterdam, Meibergdreef 9, 1105 Amsterdam, The Netherlands; 2 Department of Pediatric Neurology, Brain Center, University Medical Center, Utrecht, The Netherlands; 3 Department of Pathology, University Medical Center Utrecht, Utrecht, The Netherlands; 4 Department of Pathology and Medical Biology, University Medical Center Groningen, University of Groningen, Groningen, The Netherlands; 5 Department of Neuropathology, University Hospital Erlangen, Erlangen, Germany; 6 Institute of Neuropathology, University Hospital Münster, Münster, Germany; 7 Department of Pediatrics, Medical University of Vienna, Vienna, Austria; 8 Division of Neuropathology and Neurochemistry, Department of Neurology, Medical University of Vienna, Vienna, Austria; 9 Department of Neurology and Epileptology, Children’s Memorial Health Institute, Warsaw, Poland; 10 Department of Child Neurology, Medical University of Warsaw, Warsaw, Poland; 11 Department of Pathology, Children’s Memorial Health Institute, Warsaw, Poland; 12 Pathology Unit, Anna Meyer Children’s Hospital, Florence, Italy; 13 Department of Neurosurgery, Anna Meyer Children’s Hospital, Florence, Italy; 14 Department of Pathology, Faculty of Medicine, Hacettepe University, Ankara, Turkey; 15 Laboratory of Neuropathology, Department of Neurology, Hospital de Santa Maria (CHULN), Lisbon, Portugal; 16 Hopp Children’s Cancer Center Heidelberg (KiTZ), Heidelberg, Germany; 17 Pediatric Glioma Research Group, German Cancer Research Center (DKFZ), Heidelberg, Germany; 18 Center for Experimental & Molecular Medicine and Department of Clinical Epidemiology, Biostatistics & Bioinformatics, Amsterdam UMC, University of Amsterdam, Amsterdam, The Netherlands; 19 Princess Máxima Center for Pediatric Oncology, Utrecht, The Netherlands; 20 Department of Pediatric Oncology, Emma Children’s Hospital, Amsterdam UMC, University of Amsterdam, Amsterdam, The Netherlands; 21 Stichting Epilepsie Instellingen Nederland (SEIN), Heemstede, The Netherlands

**Keywords:** SEGA, TSC, Methylation, RNA-sequencing, Low-grade glioma

## Abstract

Tuberous sclerosis complex (TSC) is a monogenic disorder caused by mutations in either the *TSC1* or *TSC2* gene, two key regulators of the mechanistic target of the rapamycin complex pathway. Phenotypically, this leads to growth and formation of hamartomas in several organs, including the brain. Subependymal giant cell astrocytomas (SEGAs) are low-grade brain tumors commonly associated with TSC. Recently, gene expression studies provided evidence that the immune system, the MAPK pathway and extracellular matrix organization play an important role in SEGA development. However, the precise mechanisms behind the gene expression changes in SEGA are still largely unknown, providing a potential role for DNA methylation. We investigated the methylation profile of SEGAs using the Illumina Infinium HumanMethylation450 BeadChip (SEGAs *n* = 42, periventricular control *n* = 8). The SEGA methylation profile was enriched for the adaptive immune system, T cell activation, leukocyte mediated immunity, extracellular structure organization and the ERK1 & ERK2 cascade. More interestingly, we identified two subgroups in the SEGA methylation data and show that the differentially expressed genes between the two subgroups are related to the MAPK cascade and adaptive immune response. Overall, this study shows that the immune system, the MAPK pathway and extracellular matrix organization are also affected on DNA methylation level, suggesting that therapeutic intervention on DNA level could be useful for these specific pathways in SEGA. Moreover, we identified two subgroups in SEGA that seem to be driven by changes in the adaptive immune response and MAPK pathway and could potentially hold predictive information on target treatment response.

**Supplementary Information:**

The online version contains supplementary material available at 10.1007/s10571-021-01157-5.

## Background

Tuberous sclerosis complex (TSC) is a multisystem monogenetic disorder caused by mutations in either *TSC1* or *TSC2* and is characterized by hamartoma development in several organs, including the brain, kidneys, lungs, heart, eyes, and skin (Curatolo et al. 2008). Patients with TSC often have neurological manifestations including neurodevelopmental disorders (such as autism) and severe epilepsy (Curatolo et al. 2015). The majority of patients with TSC have seizure onset before the age of two (Davis et al. 2017). The hallmark brain lesions in TSC include cortical/subcortical tubers, subependymal nodules (SENs) and subependymal giant cell astrocytomas (SEGAs) (Aronica et al. 2012; Aronica and Crino 2014). SEGAs are benign, slow growing tumors classified as WHO grade I and make up 1–2% of all paediatric brain tumors (Jozwiak et al. 2015; Louis et al. 2016). Usually, SEGAs develop during the first two decades of life in patients with TSC, with a mean age at presentation below 18 years (Jozwiak et al. 2015; Adriaensen et al. 2009). They are typically located near the foramen of Monro where extended growth of the tumor can result in blockage of the cerebral fluid circulation and subsequent obstructive hydrocephalus (Cuccia et al. 2003). SEGAs are thought to arise from SEN along the ependymal lining of the lateral ventricles (Buccoliero et al. 2009; Fujiwara et al. 1989; Morimoto and Mogami 1986). Histologically, they are characterized by spindle cells, gemistocytic-like cells and giant cells and demonstrate an immature neuroglial phenotype.

Tumor suppressors hamartin (*TSC1*) and tuberin (*TSC2*) can form an intracellular complex with TBC1 domain family member 7 (TBC1D7) that exerts GTPase-activating protein (GAP) activity towards the small GTPase Ras homolog enriched in brain 1 (RHEB1) (Dibble et al. 2012; Inoki et al. 2003). Inhibition of RHEB1 is important in regulating the mechanistic target of rapamycin complex (mTOR) pathway, which can affect cell growth and proliferation. Pathogenic loss of function mutations in *TSC1* or *TSC2* result in constitutive activation of the mTOR pathway and uncontrolled cell cycle progression (Chan et al. 2004). Besides the mTOR pathway, the immune system, the mitogen-activated protein kinase (MAPK) pathway and extracellular matrix (ECM) organization have been suggested to play a role in SEGA development based on gene expression studies (Bongaarts et al. 2019; Martin et al. 2017; Tyburczy et al. 2010). However, the precise mechanisms behind these gene expression changes in SEGA are still largely unknown.

Gene expression can be controlled through regulation of the epigenome, via epigenetic mechanisms (Keshet et al. 1985). DNA methylation is one of most recognized epigenetic markers, generally associated with silencing of gene expression, and its role in tumorigenesis has become a topic of interest (Klutstein et al. 2016). It is characterized by the addition of a methyl or hydroxymethyl by DNA methyltransferases (DNMTs) to cytosine residues in CG (CpG sites), CXG and CXX DNA sequences (where X corresponds to A, T, or C). Changes in DNA methylation have been well studied in cancer including central nervous system (CNS) tumors (Binder et al. 2019; Jones and Baylin 2007) and profound changes of methylation profiles have also been seen in neuro-psychiatric diseases such as autism spectrum disorders, epilepsy and TSC (Martin et al. 2017; Henshall and Kobow 2015; Gos 2013). Furthermore, DNA methylation profiling is highly robust and reproducible and has therefore been successfully used to distinguish subtypes in CNS tumors and focal cortical dysplasia (Laffaire et al. 2011; Kobow et al. 2019; Capper et al. 2018a,b; Stone et al. 2018). These DNA methylation-based classifications of CNS tumors have proven helpful for better diagnostics especially in cases with ambiguous histology or contradictory molecular profiles. Although, SEGAs have been included in previous methylation-based studies, none of these studies have performed an in-depth exploration of the molecular contribution of DNA methylation in SEGAs (Martin et al. 2017; Capper et al. 2018a,b; Capper et al. 2018a,b). Therefore, in this study, we aimed to identify distinct methylation patterns and pathways that might contribute to SEGA pathogenesis by performing a comprehensive analysis of genomic DNA methylation patterns in SEGAs.

## Results

### The Methylation Profile of SEGAs

**Fig. 1:**
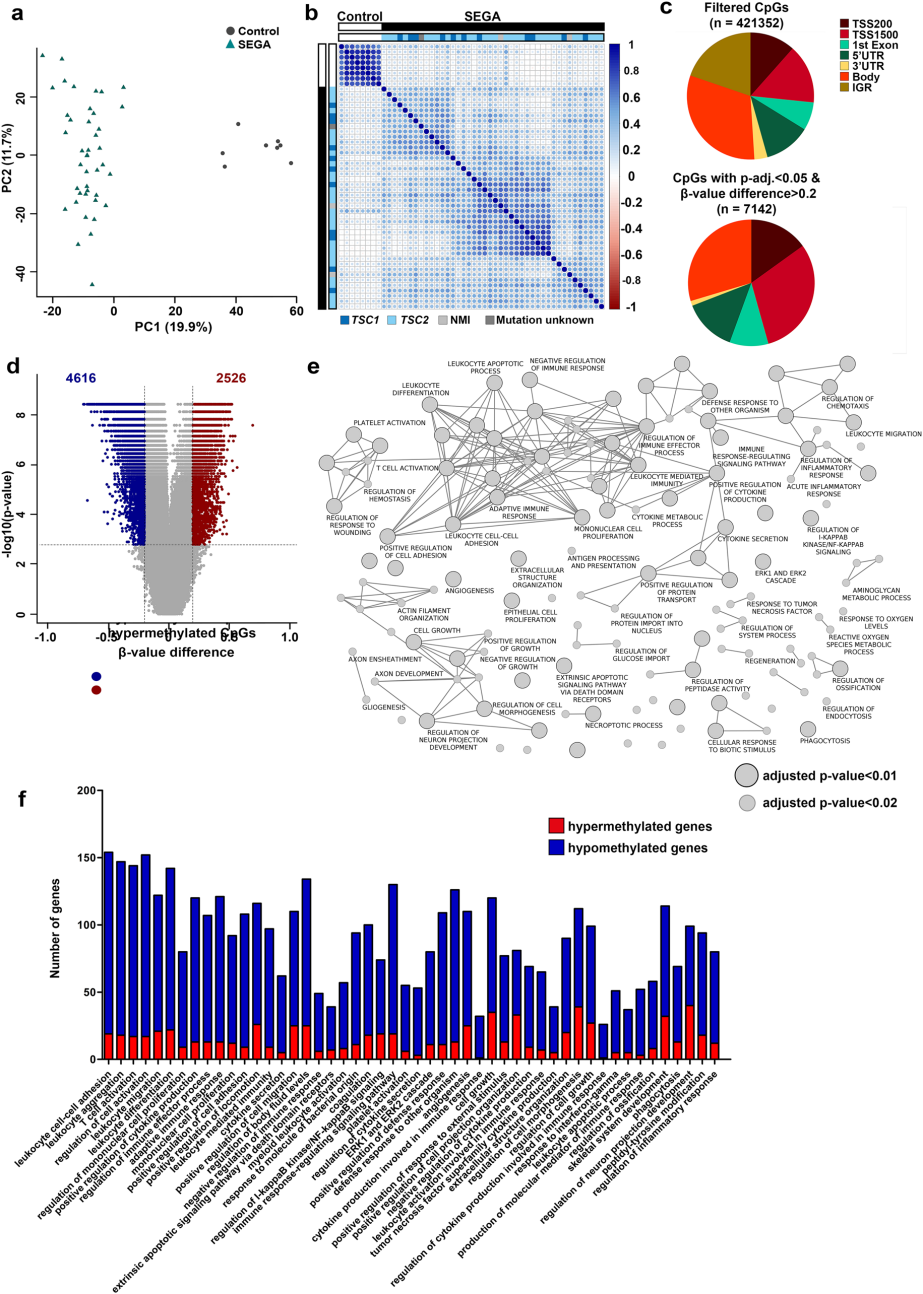
The methylation profile of SEGAs. **a** A principal component analysis (PCA) of the methylation data in SEGA (*n* = 42) and periventricular control tissue (*n* = 8) showing that the major source of variability in CpG methylation was the diagnosis. *x*-axis: the first principal component (PC); *y*-axis: the second PC. **b** Spearman’s rank correlation matrix of the methylation data showing separate clustering of SEGAs from periventricular control tissue. The scale bar indicates the strength of the correlation with 1 indicating a strong positive correlation (dark blue) and − 1 indicating a negative correlation (dark red) between samples. **c** Pie charts showing the distribution of CpGs on the gene region (TSS200, TSS1500, 5’UTR and Exon 1, IGR, 3’UTR or gene body). The upper pie chart shows the distribution for 421,352 CpGs selected after filtering for probes with a detection *p*-values of more than 0.01, located on the sex chromosomes, or in SNPs were removed as well as cross-hybridization probes. The lower pie chart shows the gene distribution after selecting for an adjusted *p*-value < 0.01 and a *β*-value difference of > 0.2. **d** Volcano plot showing the differentially methylated CpGs on the TSS-associated regions (adjusted *p*-value < 0.01 and a *β*-value difference of > 0.2) between SEGAs and control tissue. A total of 4616 CpGs were hypomethylated and 2526 were hypermethylated in SEGA compared to control tissue. **e** Schematic overview using Cytoscape of GO terms enriched in SEGA compared to control tissue (adjusted *p*-value < 0.02). Lines indicate genes in common between GO terms.** f** Graphical representation of hypermethylated (red) and hypomethylated (blue) in the top 50 GO terms

To characterize the methylation profile of SEGAs, DNA was extracted from SEGA samples and control brain samples and analyzed using the 450k methylation array. In total, 42 SEGA samples were included from 39 TSC patients and 3 patients with no other manifestations of TSC (surgical specimens) and 8 location-matched periventricular controls (autopsy specimens; see materials and methods and Table 1). A total of 421,352 CpGs were analyzed with a principal component analysis (PCA) indicating that the major source of variability was the diagnosis (SEGA or control; Fig. 1a & Supplementary Fig. 2), which was confirmed with a spearman’s correlation matrix using the top 5% most variable CpGs (Fig. 1b). Furthermore, no specific clustering was seen based on the TSC mutation (Fig. 1b). To further assess other potential confounders on the methylation profile a principal variance component analysis (PVCA) was performed, showing that the major contributor to the variance between the samples was again the diagnosis (Supplementary Fig. 1a).

**Table 1:** Summary of clinicopathological features of patients with SEGA and control tissue

Sample	Diagnosis	Age (at surgery)^c^	Gender	Mutation	Subgroup based on methylation	Location of tumour	Size (mm)	Tumour regrowth	Epilepsy	Age of epilepsy onset (months)	Seizure frequency	AED	Type of AED	mTOR inhibitors	Other clinical manifestations
S1^a^	SEGA	43	Female	*TSC2*	SEGA2b	Ventricle	19	No	yes	324	Yearly	Yes	Valproic acid, carbamazepine	Yes	Tubers, SEN, angiomyolipoma
S2^a,b^	SEGA	7	Male	*TSC1*	SEGA1	Third ventricle	7	No	Yes	10	Daily	Yes	Vigabatrin	No	Tubers, SEN, rhabdomyoma, learning impairment
S3^a,b^	SEGA	36	Female	NMI	SEGA2a	Ventricle	Unknown	No	Yes	12	Unknown	Unknown	Unknown	Unknown	Angiomyolipoma
S4^a^	SEGA	47	Male	*TSC1*	SEGA2b	Ventricle	Unknown	No	No	N/A	N/A	N/A	N/A	N/A	Angiomyolipoma
S5^a,b^	SEGA	11	Female	*TSC2*	SEGA1	Ventricle	Unknown	No	No	N/A	N/A	N/A	N/A	N/A	No other signs of TSC
S6^a,b^	SEGA	16	Male	*TSC1*	SEGA1	Ventricle	28	No	No	N/A	N/A	N/A	N/A	N/A	Tubers, mild angiofibroma
S7^a,b^	SEGA	5	Female	*TSC2*	SEGA1	Third ventricle	66	No	Yes	1	Daily	Yes	Vigabatrin, valproic acid,topiramate,carbamazepine, Adrenocorticotropic hormone,levetiracetam, phenytoin	No	Tubers, angiomyolipoma, autism
S8^a,b^	SEGA	10	Male	*TSC2*	SEGA1	Ventricle	60	No	Yes	3	No	Yes	Vigabatrin, valproic acid,topiramate	No	Tubers, angiomyolipoma, autism
S9^a,b^	SEGA	8	Male	*TSC2*	SEGA1	Ventricle	31	No	Yes	4	No	Yes	Vigabatrin, valproic acid	No	Tubers, Angiomyolipoma
S10^a,b^	SEGA	4	Male	*TSC2*	SEGA1	Ventricle	18	No	Yes	3	Daily	Yes	Vigabatrin,valproic acid,lamotrigine,oxcarbazepine	No	Tubers, angiomyolipoma, autism
S11^a^	SEGA	28	Female	*TSC2*	SEGA2a	Foramen monro	12	No	Yes	204	Monthly	Yes	Unknown	No	Angiomyolipoma, learning impairment
S12^a^	SEGA	32	Male	*TSC2*	SEGA2b	Third ventricle	21	No	Yes	372	Unknown	Yes	Unknown	No	Adenoma sebaceum
S13^a,b^	SEGA	6	Male	*TSC2*	SEGA2a	Ventricle	Unknown	Yes	Unknown	Unknown	Unknown	Unknown	Unknown	Unknown	Numerous white spots at the back
S14^a,b^	SEGA	26	Female	*TSC2*	SEGA2a	Ventricle	14	No	Unknown	Unknown	Unknown	Unknown	Unknown	Unknown	Suspected cardial Rhabdomyoma, multiple hypopigmentations, Angiofibroma
S15^a^	SEGA	53	Female	*TSC1*	SEGA2a	Foramen monro	Unknown	No	Unknown	Unknown	Unknown	Unknown	Unknown	No	Tubers
S16^a^	SEGA	23	Female	*TSC2*	SEGA2a	Ventricle	Unknown	No	Yes	Unknown	Unknown	Yes	VP-Shunt, carbamazepine, gabapentin	No	Tubers
S17^a^	SEGA	13	Male	*TSC1*	SEGA1	Ventricle	Unknown	No	Unknown	Unknown	Unknown	Unknown	Unknown	No	Tubers
S18^a,b^	SEGA	13	Male	*TSC2*	SEGA2b	Caudate nucleus	20	No	Yes	6	Weekly	Yes	Topiramate	Yes	Tubers, SEN, minor psychomotor delay
S19^a,b^	SEGA	8	Male	NMI	SEGA2b	Caudate nucleus	30	No	Yes	12	Weekly	Yes	Phenobarbital, Carbamazepine	No	Minor psychomotor delay
S20^a^	SEGA	17	Male	*TSC2*	SEGA2a	Ventricle	40	No	No	N/A	N/A	N/A	N/A	N/A	No other signs of TSC
S21^a,b^	SEGA	13	Female	*TSC1*	SEGA2a	Ventricle	40	No	Yes	6	Daily	Yes	Clonazepam, carbamazepine, valproic acid	No	Tubers, minor psychomotor delay
S22^a^	SEGA	9	Male	*TSC1*	SEGA2b	Caudate nucleus	30	No	Yes	24	Monthly	Yes	Carbamazepine	No	No other signs of TSC
S23^a,n^	SEGA	22	Male	*TSC2*	SEGA2a	Ventricle	30	No	No	N/A	N/A	N/A	N/A	N/A	Tubers
S24^a,b^	SEGA	19	Male	*TSC1*	SEGA2a	Foramen Monro	30	No	No	N/A	N/A	N/A	N/A	N/A	Tubers
S25^a,b^	SEGA	20	Male	*TSC2*	SEGA1	Ventricle	45	No	Yes	4	Weekly	Yes	Clonazepam, carbamazepine, phenytoin	No	Tubers, SEN, white matter changes, angiofibroma
S26^a,b^	SEGA	8	Male	*TSC2*	SEGA2a	Frontal operculum	45	No	Yes	2	Daily	Yes	Lamotrigine, oxcarbazepine	No	Tubers, calcifications, white matter changes
S27^a,b^	SEGA	1	Female	*TSC2*	SEGA2a	Ventricle	20	Yes	No	N/A	N/A	N/A	N/A	N/A	Tubers
S28^a^	SEGA	27	Male	*TSC1*	SEGA2a	Ventricle	50	No	Yes	12	Weekly	Yes	Valproic acid, phenobarbital, carbamazepine	No	Tubers
S29^a,b^	SEGA	9	Female	*TSC2*	SEGA2b	Ventricle	Unknown	Unknown	Yes	Unknown	Unknown	Unknown	Unknown	Unknown	Unknown
S30^a,b^	SEGA	13	Female	*TSC2*	SEGA1	Ventricle	24	No	Yes	120	Unknown	Yes	Carbamazepine	No	Tubers, SEN, Angiomyolipoma, learning impairment
S31^a^	SEGA	15	Male	*TSC1*	SEGA2b	Ventricle	15	No	Yes	60	Daily	Yes	Carbamazepine	No	Tubers, angiomyolipoma
S32^a,b^	SEGA	28	Male	*TSC2*	SEGA2a	Ventricle	34	Yes	Yes	84	Daily	Yes	Carbamazepine, levetiracetam	No	Unknown
S33^a,b^	SEGA	1	Male	*TSC2*	SEGA2a	Ventricle	30	No	Yes	1	Daily	Yes	Clonazepam, valproic acid, phenytoin, rufinamide, carbamazepine	Yes	Multiple SEGAs, tubers, drug resistant epilepsy
S34^a,b^	SEGA	1	Female	*TSC2*	SEGA2a	Ventricle	30	No	Yes	1	Daily	Yes	Vigabatrin	No	Multiple SEGAs, tubers, drug-resistant epilepsy
S35^a,b^	SEGA	23	Male	*TSC1*	SEGA2a	Third ventricle	Unknown	No	Yes	Unknown	Unknown	Unknown	Unknown	Unknown	Mental retardation, Angiofibroma
S36^a,b^	SEGA	23	Male	*TSC2*	SEGA2a	Foramen monro	Unknown	Yes	Yes	36	Yearly	Yes	Carbamazepine, phenytoin	No	Tubers, angiomyolipoma
S37^a^	SEGA	14	Male	*TSC2*	SEGA2a	Ventricle	40	No	Yes	48	Unknown	Yes	Carbamazepine	No	Tubers, SEN, angiomyolipoma
S38^a^	SEGA	19	Male	*TSC2*	SEGA2b	Foramen monro	12	No	Yes	Unknown	Unknown	Unknown	Unknown	Unknown	Tubers
S39^a^	SEGA	28	Male	*TSC2*	SEGA2b	Foramen monro	25	Yes	Yes	Unknown	Unknown	Unknown	Unknown	Unknown	Tubers
S40^a^	SEGA	22	Male	*TSC2*	SEGA2b	Foramen monro	40	No	Yes	Unknown	Unknown	Unknown	Unknown	Unknown	Tubers
S41^a^	SEGA	13	Female	*TSC2*	SEGA1	Caudate nucleus	30	Yes	Yes	6	Daily	Yes	Vigabatrin, levetiracetam	Yes	Tubers, drug-resistant epilepsy, minor psychomotor delay
S42^a^	SEGA	7	Male	Unknown	SEGA1	Caudate nucleus	40	No	No	N/A	N/A	N/A	N/A	N/A	Minor psychomotor delay
S43^b^	SEGA	10	Male	*TSC2*	ND	Ventricle	42	No	Yes	36	Daily	Yes	Lamotrigine, Valproic acid, Pipamperone	No	Unknown
S44^b^	SEGA	24	Male	*TSC1*	ND	Ventricle	40	No	Yes	72	Daily	Yes	Phenytoin	No	Unknown
S45^b^	SEGA	7	Female	*TSC2*	ND	Ventricle	45	No	Yes	5	Daily	Yes	Levetiracetam, carbamazepine, oxcarbazepine	No	Unknown
S46^b^	SEGA	10	Male	*TSC2*	ND	Ventricle	45	No	Yes	12	Daily	Yes	Vigabatrin, pyridoxine, lamotrigine, valproic acid	No	Unknown
S47^b^	SEGA	9	Female	*TSC1*	ND	Third ventricle	30	No	No	N/A	N/A	N/A	N/A	No	Unknown
S48^b^	SEGA	15	Female	*TSC2*	ND	Foramen monro	10	No	Yes	6	Daily	Unknown	Unknown	Unknown	Angiomyolipoma, liver cysts, kidney cyst, rhabdomyoma
S49^b^	SEGA	4	Female	*TSC1*	ND	Ventricle	Unknown	No	Yes	24	Daily	Yes	Valproic acid, frisium, carbamazepine	No	Tubers, numerous white spots at the back, minor psychomotor delay, autism
S50^b^	SEGA	3	Female	*TSC2*	ND	Caudate nucleus	20	Yes	Yes	4	Weekly	Yes	Vigabatrin, topiramate	No	Tubers, SEN
S51^b^	SEGA	17	Female	*TSC2*	ND	Ventricle	27	No	No	N/A	N/A	N/A	N/A	N/A	No other signs of TSC
S52^b^	SEGA	13	Male	*TSC2*	ND	Ventricle	20	Yes	Yes	4	Weekly	Yes	Vigabatrin,valproic acid,lamotrigine,ketogenic diet, levetiracetam, topiramate, vagus nerve stimulation	No	Tubers
S53^b^	SEGA	15	Female	NMI	ND	Frontal lobe, interhemispheric	50	No	No	N/A	N/A	N/A	N/A	No	Tubers, mental retardation
S54^b^	SEGA	15	Male	*TSC1*	ND	Ventricle	33	No	Yes	168	Monthly	Yes	Pregabalin, Oxcarbazepine	No	Tubers
S55^b^	SEGA	24	Male	*TSC2*	ND	Third ventricle	22	No	No	N/A	N/A	N/A	N/A	No	Tubers, SEN
C1^a,b^	Control	2 months	Female	Control	Control	Periventricular	N/A	N/A	N/A	N/A	N/A	N/A	N/A	N/A	N/A
C2^a,b^	Control	13	Male	Control	Control	Periventricular	N/A	N/A	N/A	N/A	N/A	N/A	N/A	N/A	N/A
C3^a,b^	Control	7	Female	Control	Control	Periventricular	N/A	N/A	N/A	N/A	N/A	N/A	N/A	N/A	N/A
C4^a,b^	Control	2	Male	Control	Control	Periventricular	N/A	N/A	N/A	N/A	N/A	N/A	N/A	N/A	N/A
C5^a^	Control	17	Female	Control	Control	Periventricular	N/A	N/A	N/A	N/A	N/A	N/A	N/A	N/A	N/A
C6^a^	Control	15	Male	Control	Control	Periventricular	N/A	N/A	N/A	N/A	N/A	N/A	N/A	N/A	N/A
C7^a,b^	Control	44	Female	Control	Control	Periventricular	N/A	N/A	N/A	N/A	N/A	N/A	N/A	N/A	N/A
C8^a^	Control	56	Male	Control	Control	periventricular	N/A	N/A	N/A	N/A	N/A	N/A	N/A	N/A	N/A
C9^b^	Control	1	Female	Control	Control	Periventricular	N/A	N/A	N/A	N/A	N/A	N/A	N/A	N/A	N/A
C10^b^	Control	3 months	Female	Control	Control	Periventricular	N/A	N/A	N/A	N/A	N/A	N/A	N/A	N/A	N/A
C11^b^	Control	17	Female	Control	Control	Periventricular	N/A	N/A	N/A	N/A	N/A	N/A	N/A	N/A	N/A

*AED* antiepileptic drugs, *SEN* subependymal nodule, *NMI* no mutation identified, *ND* not defined as this sample was not included in the methylation analysis

^a^SEGA samples used for methylation analysis

^b^Immunohistochemistry

^c^Age at surgery is the same as the age at tissue collection

Since the majority of the differentially methylated CpGs (adjusted *p*-value 0.01, *β*-value difference of > 0.2) were located at the TSS-associated regions (Fig. 1c), we narrowed our data set to these CpGs and found 4616 CpGs hypomethylated and 2526 hypermethylated in SEGA compared to controls (adjusted *p*-value 0.01, *β*-value difference of > 0.2, TSS-associated regions; Fig. 1d). The 7142 differentially methylated CpGs were located on 3875 genes. We identified 227 enriched GO terms (adjusted *p*-value < 0.05) for these genes (Fig. 1e; Table 2) including adaptive immune system, T cell activation, leukocyte mediated immunity, extracellular structure organization and the ERK1 & ERK2 cascade. When accounting for the number of probes in each gene (using missMethyl) we found that the adaptive immune system, T cell activation, leukocyte mediated immunity, the ERK1 & ERK2 cascade and the extracellular matrix were still among the enriched GO terms (Supplementary Table 1). The majority of the enriched GO terms contained more hypomethylated genes then hypermethylated genes (Fig. 1f; Table 2).

**Table 2:** GO terms enriched in SEGA compared to control tissue

GO term	GO	GeneRatio	BgRatio	*p* value	p.adjust	*q* value	Count	Hypo	Hyper	Both hypo and hyper methylated
Leukocyte cell–cell adhesion	GO:0007159	163/2795	476/16672	4.05218E-21	1.28302E-17	9.57805E-18	163	19	135	9
Leukocyte aggregation	GO:0070486	155/2795	444/16672	4.59205E-21	1.28302E-17	9.57805E-18	155	18	129	8
T cell activation	GO:0042110	152/2795	436/16672	1.30039E-20	1.81665E-17	1.35617E-17	152	17	127	8
Regulation of cell activation	GO:0050865	160/2795	482/16672	3.05025E-19	2.8408E-16	2.12073E-16	160	17	135	8
Leukocyte migration	GO:0050900	129/2795	362/16672	1.441E-18	1.15033E-15	8.58752E-16	129	21	101	7
Regulation of mononuclear cell proliferation	GO:0032944	81/2795	186/16672	6.18246E-18	3.74405E-15	2.79502E-15	81	9	71	1
Leukocyte differentiation	GO:0002521	150/2795	454/16672	6.70016E-18	3.74405E-15	2.79502E-15	150	22	120	8
Positive regulation of cytokine production	GO:0001819	128/2795	376/16672	1.33813E-16	5.75189E-14	4.29393E-14	128	13	107	8
Regulation of immune effector process	GO:0002697	111/2795	310/16672	2.52134E-16	1.00637E-13	7.51283E-14	111	13	94	4
Adaptive immune response	GO:0002250	124/2795	369/16672	1.23676E-15	4.31938E-13	3.22452E-13	124	13	108	3
Mononuclear cell proliferation	GO:0032943	93/2795	248/16672	2.5813E-15	8.01351E-13	5.98228E-13	93	12	80	1
Positive regulation of cell adhesion	GO:0045785	121/2795	371/16672	3.21282E-14	7.82098E-12	5.83855E-12	121	9	99	13
Positive regulation of locomotion	GO:0040017	125/2795	411/16672	3.17827E-12	4.97716E-10	3.71557E-10	125	26	90	9
Leukocyte-mediated immunity	GO:0002443	100/2795	305/16672	3.88427E-12	5.5961E-10	4.17763E-10	100	9	88	3
Cytokine secretion	GO:0050663	64/2795	162/16672	3.90565E-12	5.5961E-10	4.17763E-10	64	5	57	2
Positive regulation of cell migration	GO:0030335	118/2795	386/16672	8.95329E-12	1.16351E-09	8.6859E-10	118	25	85	8
Regulation of body fluid levels	GO:0050878	142/2795	495/16672	1.34247E-11	1.7006E-09	1.26954E-09	142	25	109	8
Negative regulation of immune response	GO:0050777	50/2795	115/16672	1.39992E-11	1.7006E-09	1.26954E-09	50	6	43	1
Extrinsic apoptotic signaling pathway via death domain receptors	GO:0008625	40/2795	84/16672	4.96589E-11	5.33642E-09	3.98377E-09	40	7	32	1
Myeloid leukocyte activation	GO:0002274	59/2795	152/16672	6.24731E-11	6.58679E-09	4.9172E-09	59	8	49	2
Response to molecule of bacterial origin	GO:0002237	98/2795	311/16672	8.11855E-11	8.4012E-09	6.2717E-09	98	11	83	4
Coagulation	GO:0050817	107/2795	354/16672	1.75366E-10	1.71921E-08	1.28343E-08	107	18	82	7
Regulation of I-kappaB kinase/NF-kappaB signaling	GO:0043122	78/2795	231/16672	1.87373E-10	1.80524E-08	1.34766E-08	78	19	55	4
Immune response-regulating signaling pathway	GO:0002764	137/2795	490/16672	2.15352E-10	2.03963E-08	1.52264E-08	137	19	111	7
Platelet activation	GO:0030168	60/2795	161/16672	2.96605E-10	2.7171E-08	2.02838E-08	60	6	49	5
Regulation of cytokine secretion	GO:0050707	55/2795	142/16672	3.02988E-10	2.7308E-08	2.03861E-08	55	3	50	2
ERK1 and ERK2 cascade	GO:0070371	82/2795	251/16672	4.065E-10	3.49465E-08	2.60884E-08	82	11	69	2
Positive regulation of defense response	GO:0031349	114/2795	396/16672	1.13884E-09	9.09119E-08	6.78679E-08	114	11	98	5
Defense response to other organism	GO:0098542	131/2795	475/16672	1.40194E-09	1.10339E-07	8.23707E-08	131	13	113	5
Angiogenesis	GO:0001525	117/2795	411/16672	1.43172E-09	1.11117E-07	8.29517E-08	117	25	85	7
Cytokine production involved in immune response	GO:0002367	34/2795	72/16672	1.82332E-09	1.3585E-07	1.01415E-07	34	1	31	2
Positive regulation of response to external stimulus	GO:0032103	82/2795	261/16672	3.27412E-09	2.37179E-07	1.7706E-07	82	13	64	5
Cell growth	GO:0016049	131/2795	481/16672	3.31066E-09	2.37179E-07	1.7706E-07	131	35	85	11
Positive regulation of cell projection organization	GO:0031346	90/2795	296/16672	3.54321E-09	2.50626E-07	1.87099E-07	90	33	48	9
Negative regulation of cytokine production	GO:0001818	70/2795	212/16672	4.62996E-09	3.1941E-07	2.38447E-07	70	9	60	1
Leukocyte activation involved in immune response	GO:0002366	70/2795	214/16672	7.201E-09	4.84809E-07	3.61922E-07	70	7	58	5
Tumor necrosis factor superfamily cytokine production	GO:0071706	43/2795	109/16672	1.3446E-08	8.53818E-07	6.37396E-07	43	5	34	4
Extracellular structure organization	GO:0043062	96/2795	331/16672	1.55009E-08	9.62432E-07	7.18479E-07	96	20	70	6
Regulation of cell morphogenesis	GO:0022604	124/2795	460/16672	1.67534E-08	1.02877E-06	7.67999E-07	124	39	73	12
Regulation of cell growth	GO:0001558	107/2795	382/16672	1.85673E-08	1.09215E-06	8.15315E-07	107	27	72	8
Regulation of cytokine production involved in immune response	GO:0002718	28/2795	58/16672	2.66028E-08	1.50158E-06	1.12097E-06	28	1	25	2
Response to interferon-gamma	GO:0034341	55/2795	159/16672	3.39595E-08	1.79074E-06	1.33683E-06	55	5	46	4
Leukocyte apoptotic process	GO:0071887	39/2795	97/16672	3.3969E-08	1.79074E-06	1.33683E-06	39	5	32	2
Production of molecular mediator of immune response	GO:0002440	54/2795	156/16672	4.39E-08	2.27142E-06	1.69567E-06	54	3	49	2
Regulation of ossification	GO:0030278	59/2795	177/16672	5.0274E-08	2.53091E-06	1.88939E-06	59	8	50	1
Skeletal system development	GO:0001501	127/2795	483/16672	5.41576E-08	2.65467E-06	1.98178E-06	127	32	82	13
Phagocytosis	GO:0006909	73/2795	238/16672	7.04139E-08	3.36302E-06	2.51057E-06	73	13	56	4
Regulation of neuron projection development	GO:0010975	108/2795	398/16672	9.70394E-08	4.48146E-06	3.34552E-06	108	40	59	9
Peptidyl-tyrosine modification	GO:0018212	99/2795	358/16672	1.23872E-07	5.49362E-06	4.10112E-06	99	18	76	5
Regulation of inflammatory response	GO:0050727	85/2795	295/16672	1.39894E-07	6.10724E-06	4.55921E-06	85	12	68	5
Positive regulation of secretion	GO:0051047	95/2795	346/16672	3.16778E-07	1.18802E-05	8.86889E-06	95	15	75	5
Mesenchymal cell differentiation	GO:0048762	58/2795	183/16672	4.5348E-07	1.62439E-05	1.21265E-05	58	14	42	2
Regulation of peptidase activity	GO:0052547	103/2795	386/16672	4.59093E-07	1.62584E-05	1.21373E-05	103	16	85	2
Regulation of leukocyte apoptotic process	GO:2000106	32/2795	79/16672	4.62614E-07	1.62584E-05	1.21373E-05	32	4	26	2
Negative regulation of response to external stimulus	GO:0032102	74/2795	257/16672	9.0766E-07	2.91495E-05	2.17608E-05	74	12	56	6
Regulation of chemotaxis	GO:0050920	56/2795	178/16672	9.26622E-07	2.95884E-05	2.20884E-05	56	11	41	4
Epithelial cell proliferation	GO:0050673	91/2795	336/16672	1.06365E-06	3.33916E-05	2.49276E-05	91	17	68	6
Necroptotic process	GO:0070266	16/2795	28/16672	1.48267E-06	4.47114E-05	3.33781E-05	16	3	13	0
Regulation of response to biotic stimulus	GO:0002831	44/2795	130/16672	1.51917E-06	4.53964E-05	3.38895E-05	44	7	35	2
Cellular response to biotic stimulus	GO:0071216	54/2795	172/16672	1.54582E-06	4.57039E-05	3.4119E-05	54	5	47	2
Positive regulation of protein transport	GO:0051222	113/2795	444/16672	1.66434E-06	4.86928E-05	3.63504E-05	113	17	87	9
Regulation of multi-organism process	GO:0043900	96/2795	363/16672	1.71017E-06	4.97731E-05	3.71568E-05	96	18	74	4
Regulation of response to wounding	GO:1903034	47/2795	143/16672	1.74515E-06	5.00883E-05	3.73921E-05	47	5	38	4
Regulation of leukocyte differentiation	GO:1902105	67/2795	231/16672	2.21428E-06	6.12544E-05	4.57279E-05	67	6	55	6
Positive regulation of peptidase activity	GO:0010952	48/2795	149/16672	2.58245E-06	7.00522E-05	5.22957E-05	48	4	44	0
Negative regulation of locomotion	GO:0040013	70/2795	248/16672	3.84631E-06	9.81424E-05	7.32657E-05	70	13	53	4
Negative regulation of defense response	GO:0031348	46/2795	143/16672	4.32573E-06	0.000109609	8.18257E-05	46	7	38	1
Actin filament organization	GO:0007015	86/2795	323/16672	4.36918E-06	0.000109609	8.18257E-05	86	15	61	10
Axon development	GO:0061564	110/2795	439/16672	4.93975E-06	0.000121067	9.03797E-05	110	41	64	5
Circulatory system process	GO:0003013	120/2795	488/16672	4.96994E-06	0.000121275	9.05349E-05	120	28	84	8
Acute inflammatory response	GO:0002526	43/2795	132/16672	6.11633E-06	0.000143004	0.000106756	43	3	37	3
Regulation of osteoblast differentiation	GO:0045667	37/2795	109/16672	9.3096E-06	0.000207259	0.000154724	37	6	30	1
Response to tumor necrosis factor	GO:0034612	73/2795	268/16672	9.62533E-06	0.000212594	0.000158707	73	12	61	0
Cellular response to tumor necrosis factor	GO:0071356	69/2795	250/16672	1.03519E-05	0.000226848	0.000169348	69	10	59	0
Regulation of actin filament-based process	GO:0032970	83/2795	317/16672	1.26174E-05	0.000268083	0.000200131	83	15	60	8
Production of molecular mediator involved in inflammatory response	GO:0002532	19/2795	42/16672	1.51046E-05	0.000314943	0.000235113	19	3	15	1
Regulation of mast cell activation involved in immune response	GO:0033006	15/2795	29/16672	1.63205E-05	0.000336528	0.000251226	15	1	14	0
Regulation of mast cell degranulation	GO:0043304	15/2795	29/16672	1.63205E-05	0.000336528	0.000251226	15	1	14	0
Regulation of anatomical structure size	GO:0090066	113/2795	466/16672	1.81159E-05	0.000368115	0.000274807	113	28	79	6
Cell killing	GO:0001906	35/2795	105/16672	2.49976E-05	0.000490128	0.000365893	35	3	31	1
Response to mechanical stimulus	GO:0009612	55/2795	193/16672	2.99465E-05	0.000571483	0.000426626	55	12	41	2
Regulation of defense response to virus	GO:0050688	29/2795	82/16672	3.52341E-05	0.000649797	0.000485089	29	5	23	1
Gliogenesis	GO:0042063	63/2795	231/16672	3.65163E-05	0.000671227	0.000501088	63	23	37	3
Regulation of actin cytoskeleton organization	GO:0032956	74/2795	283/16672	3.81676E-05	0.000693508	0.000517721	74	11	56	7
Regulation of endocytosis	GO:0030100	54/2795	190/16672	3.81707E-05	0.000693508	0.000517721	54	9	43	2
Negative regulation of phosphorylation	GO:0042326	101/2795	414/16672	3.82949E-05	0.000693508	0.000517721	101	25	68	8
Response to transforming growth factor beta	GO:0071559	58/2795	209/16672	4.27717E-05	0.000749243	0.000559328	58	14	38	6
Developmental growth involved in morphogenesis	GO:0060560	57/2795	205/16672	4.67212E-05	0.000813327	0.000607168	57	22	28	7
Regulation of reactive oxygen species metabolic process	GO:2000377	44/2795	147/16672	5.0876E-05	0.00088017	0.000657069	44	6	37	1
Regulation of system process	GO:0044057	116/2795	492/16672	5.23386E-05	0.000899902	0.000671799	116	31	77	8
Regulation of coagulation	GO:0050818	30/2795	88/16672	5.75939E-05	0.000969381	0.000723667	30	3	25	2
Cell-substrate adhesion	GO:0031589	77/2795	301/16672	5.97983E-05	0.000988618	0.000738028	77	12	57	8
Single-organism carbohydrate metabolic process	GO:0044723	105/2795	439/16672	6.39186E-05	0.001041333	0.00077738	105	30	66	9
Regulation of protein localization to nucleus	GO:1900180	58/2795	212/16672	6.62976E-05	0.001076951	0.00080397	58	10	46	2
Positive regulation of NF-kappaB transcription factor activity	GO:0051092	40/2795	131/16672	6.71965E-05	0.001088389	0.000812509	40	4	35	1
Lipid phosphorylation	GO:0046834	34/2795	106/16672	7.82864E-05	0.00122539	0.000914784	34	11	21	2
Regulation of fibroblast growth factor receptor signaling pathway	GO:0040036	13/2795	26/16672	9.52236E-05	0.00144595	0.001079437	13	3	10	0
Membrane assembly	GO:0071709	13/2795	26/16672	9.52236E-05	0.00144595	0.001079437	13	2	9	2
Osteoblast differentiation	GO:0001649	55/2795	201/16672	0.000100453	0.001510051	0.00112729	55	9	43	3
Cellular response to lipid	GO:0071396	114/2795	490/16672	0.000108435	0.001620147	0.001209479	114	16	87	11
Muscle system process	GO:0003012	93/2795	385/16672	0.000112057	0.001656543	0.00123665	93	25	61	7
Cellular response to transforming growth factor beta stimulus	GO:0071560	56/2795	207/16672	0.000121675	0.001779898	0.001328737	56	14	38	4
Regulation of platelet activation	GO:0010543	14/2795	30/16672	0.000132905	0.001929851	0.001440681	14	0	12	2
Cytokine metabolic process	GO:0042107	34/2795	109/16672	0.000144766	0.002084923	0.001556446	34	1	33	0
Regulation of hemostasis	GO:1900046	28/2795	84/16672	0.000155932	0.00221155	0.001650976	28	2	24	2
Antigen processing and presentation	GO:0019882	59/2795	223/16672	0.000157968	0.002234754	0.001668299	59	11	45	3
Leukocyte homeostasis	GO:0001776	26/2795	76/16672	0.000164275	0.002278444	0.001700915	26	4	21	1
Positive regulation of cytokine biosynthetic process	GO:0042108	22/2795	60/16672	0.000164319	0.002278444	0.001700915	22	1	21	0
Leukocyte degranulation	GO:0043299	23/2795	64/16672	0.000167098	0.002305539	0.001721141	23	3	19	1
Endothelium development	GO:0003158	34/2795	110/16672	0.000176232	0.002419617	0.001806303	34	9	22	3
Detection of biotic stimulus	GO:0009595	11/2795	21/16672	0.000195504	0.002655447	0.001982356	11	0	11	0
Response to lipoprotein particle	GO:0055094	10/2795	18/16672	0.000204096	0.002715452	0.002027151	10	2	7	1
Glycoprotein metabolic process	GO:0009100	92/2795	387/16672	0.000221154	0.002900954	0.002165633	92	30	55	7
Developmental cell growth	GO:0048588	51/2795	188/16672	0.000221893	0.002903841	0.002167789	51	23	22	6
Positive regulation of cellular component biogenesis	GO:0044089	100/2795	428/16672	0.000237329	0.003077023	0.002297073	100	25	70	5
Tissue regeneration	GO:0042246	19/2795	50/16672	0.000264385	0.003388491	0.002529592	19	6	12	1
Regulation of protein import into nucleus	GO:0042306	46/2795	167/16672	0.000303369	0.003800954	0.002837505	46	7	37	2
Response to oxygen levels	GO:0070482	74/2795	301/16672	0.000307923	0.003849388	0.002873662	74	15	53	6
Aminoglycan biosynthetic process	GO:0006023	34/2795	113/16672	0.000310583	0.003873967	0.002892011	34	13	17	4
Anion transport	GO:0006820	104/2795	454/16672	0.000387883	0.004661266	0.00347975	104	39	59	6
Muscle tissue development	GO:0060537	82/2795	343/16672	0.000392014	0.004700808	0.003509268	82	18	56	8
Negative regulation of growth	GO:0045926	58/2795	226/16672	0.000420156	0.004963699	0.003705523	58	39	15	4
Regeneration	GO:0031099	44/2795	160/16672	0.000421075	0.004964068	0.003705799	44	10	29	5
Axon ensheathment	GO:0008366	32/2795	106/16672	0.000430832	0.005037194	0.003760388	32	13	16	3
Response to peptide	GO:1901652	98/2795	425/16672	0.000443414	0.005150171	0.003844729	98	23	68	7
Regulation of cell killing	GO:0031341	20/2795	56/16672	0.000479078	0.005463441	0.004078593	20	2	18	0
Cellular response to organonitrogen compound	GO:0071417	105/2795	462/16672	0.000491893	0.005541727	0.004137035	105	23	72	10
Membrane biogenesis	GO:0044091	13/2795	30/16672	0.000565032	0.006227608	0.004649062	13	2	9	2
Reactive oxygen species metabolic process	GO:0072593	58/2795	229/16672	0.000599901	0.006560166	0.004897325	58	8	49	1
Renal system process	GO:0003014	32/2795	108/16672	0.000618811	0.006714405	0.005012468	32	7	24	1
Regulation of necroptotic process	GO:0060544	7/2795	11/16672	0.000649859	0.006956727	0.005193367	7	1	6	0
Positive regulation of growth	GO:0045927	59/2795	236/16672	0.00076698	0.008010998	0.005980406	59	15	40	4
Aminoglycan metabolic process	GO:0006022	45/2795	169/16672	0.000774668	0.008031257	0.00599553	45	16	23	6
Regulation of glucose import	GO:0046324	21/2795	62/16672	0.000785601	0.008069745	0.006024262	21	6	11	4
Negative regulation of peptidase activity	GO:0010466	60/2795	242/16672	0.000868382	0.008759064	0.006538856	60	11	47	2
Response to vitamin	GO:0033273	26/2795	84/16672	0.000949639	0.009442313	0.007048918	26	1	21	4
Dorsal/ventral axis specification	GO:0009950	10/2795	21/16672	0.001011154	0.009932496	0.007414852	10	1	9	0
Lysosome localization	GO:0032418	20/2795	59/16672	0.001026036	0.010058755	0.007509107	20	2	17	1
Response to estradiol	GO:0032355	33/2795	116/16672	0.001120218	0.010682217	0.007974537	33	4	23	6
Regulation of cellular component size	GO:0032535	79/2795	340/16672	0.001201137	0.011261665	0.008407109	79	23	51	5
Viral entry into host cell	GO:0046718	32/2795	112/16672	0.001220824	0.011418088	0.008523882	32	6	25	1
Positive regulation of antigen processing and presentation	GO:0002579	8/2795	15/16672	0.001301176	0.011998307	0.00895703	8	0	7	1
Cellular response to lipoprotein particle stimulus	GO:0071402	7/2795	12/16672	0.001333899	0.012120041	0.009047907	7	1	6	0
Regulation of transmembrane transporter activity	GO:0022898	46/2795	178/16672	0.001344232	0.012157408	0.009075803	46	12	30	4
Pri-miRNA transcription from RNA polymerase II promoter	GO:0061614	12/2795	29/16672	0.001497605	0.013283522	0.009916474	12	0	11	1
Interaction with host	GO:0051701	42/2795	160/16672	0.00153631	0.013583707	0.01014057	42	7	33	2
Regulation of carbohydrate metabolic process	GO:0006109	39/2795	146/16672	0.001569464	0.013811279	0.010310458	39	11	25	3
Regulation of morphogenesis of an epithelium	GO:1905330	45/2795	175/16672	0.001676801	0.014591866	0.010893186	45	12	30	3
Regulation of cellular response to growth factor stimulus	GO:0090287	55/2795	224/16672	0.001756955	0.015221497	0.011363221	55	13	42	0
Regulation of focal adhesion assembly	GO:0051893	17/2795	49/16672	0.001793166	0.015487186	0.011561564	17	5	11	1
Cellular response to mechanical stimulus	GO:0071260	22/2795	70/16672	0.00182221	0.015689533	0.011712621	22	3	19	0
Response to corticosteroid	GO:0031960	40/2795	152/16672	0.001863875	0.015998978	0.011943629	40	9	27	4
Viral genome replication	GO:0019079	30/2795	106/16672	0.002004929	0.016857755	0.012584727	30	4	24	2
Positive regulation of lipase activity	GO:0060193	20/2795	62/16672	0.00203777	0.017097682	0.012763839	20	4	14	2
Regulation of vasoconstriction	GO:0019229	19/2795	58/16672	0.002137396	0.017773467	0.013268329	19	3	16	0
Ceramide biosynthetic process	GO:0046513	19/2795	58/16672	0.002137396	0.017773467	0.013268329	19	5	12	2
Receptor-mediated endocytosis	GO:0006898	67/2795	286/16672	0.002184578	0.018093492	0.013507235	67	14	50	3
Lipid localization	GO:0010876	79/2795	347/16672	0.002185595	0.018093492	0.013507235	79	23	50	6
Regulation of mesoderm development	GO:2000380	8/2795	16/16672	0.002221754	0.018204049	0.013589769	8	3	5	0
Negative regulation of cell projection organization	GO:0031345	38/2795	144/16672	0.002261635	0.018420414	0.01375129	38	13	21	4
Regulation of protein binding	GO:0043393	43/2795	168/16672	0.002304769	0.018494877	0.013806879	43	9	33	1
Negative regulation of hydrolase activity	GO:0051346	88/2795	394/16672	0.002306295	0.018494877	0.013806879	88	20	63	5
Hair follicle development	GO:0001942	24/2795	80/16672	0.002341939	0.018494877	0.013806879	24	7	17	0
Molting cycle process	GO:0022404	24/2795	80/16672	0.002341939	0.018494877	0.013806879	24	7	17	0
Hair cycle process	GO:0022405	24/2795	80/16672	0.002341939	0.018494877	0.013806879	24	7	17	0
Inflammatory response to antigenic stimulus	GO:0002437	15/2795	42/16672	0.002366417	0.018494877	0.013806879	15	2	13	0
Regulation of lipid storage	GO:0010883	15/2795	42/16672	0.002366417	0.018494877	0.013806879	15	4	10	1
Tolerance induction	GO:0002507	10/2795	23/16672	0.002374837	0.018494877	0.013806879	10	0	9	1
Bleb assembly	GO:0032060	6/2795	10/16672	0.002507283	0.019166483	0.014308249	6	1	3	2
Protein localization to nucleus	GO:0034504	79/2795	351/16672	0.003021511	0.022082086	0.016484818	79	13	62	4
Regulation of behavior	GO:0050795	19/2795	60/16672	0.003298011	0.02353676	0.017570768	19	6	12	1
Calcineurin-mediated signaling	GO:0097720	10/2795	24/16672	0.003466073	0.024455069	0.018256308	10	5	4	1
Response to ethanol	GO:0045471	32/2795	119/16672	0.003519758	0.024740135	0.018469117	32	6	21	5
Renal system process involved in regulation of blood volume	GO:0001977	8/2795	17/16672	0.003584492	0.024873491	0.018568671	8	0	7	1
Regulation of lipase activity	GO:0060191	23/2795	78/16672	0.003636516	0.025087472	0.018728412	23	4	17	2
Positive chemotaxis	GO:0050918	17/2795	52/16672	0.003669932	0.025255639	0.018853953	17	3	11	3
Regulation of synaptic transmission, GABAergic	GO:0032228	11/2795	28/16672	0.003804505	0.026021512	0.019425697	11	6	5	0
Sodium ion homeostasis	GO:0055078	16/2795	48/16672	0.003830346	0.026102409	0.019486088	16	2	13	1
Positive regulation of actin filament polymerization	GO:0030838	26/2795	92/16672	0.003937594	0.02668146	0.019918363	26	4	21	1
Cardiac muscle cell differentiation	GO:0055007	26/2795	92/16672	0.003937594	0.02668146	0.019918363	26	7	15	4
Regulation of glycolytic process	GO:0006110	12/2795	32/16672	0.003992027	0.027006596	0.020161085	12	4	7	1
Positive regulation of cation channel activity	GO:2001259	13/2795	36/16672	0.004061865	0.027379613	0.020439552	13	5	6	2
Regulation of developmental growth	GO:0048638	66/2795	288/16672	0.004081169	0.027410545	0.020462644	66	24	37	5
Calcium ion transport into cytosol	GO:0060402	33/2795	125/16672	0.004201336	0.028036143	0.020929668	33	7	26	0
MHC class II biosynthetic process	GO:0045342	7/2795	14/16672	0.004234521	0.028036143	0.020929668	7	1	6	0
Myoblast proliferation	GO:0051450	7/2795	14/16672	0.004234521	0.028036143	0.020929668	7	1	6	0
Regulation of ion homeostasis	GO:2000021	47/2795	193/16672	0.004288344	0.028291931	0.02112062	47	8	38	1
N-glycan processing	GO:0006491	9/2795	21/16672	0.004394081	0.028718273	0.021438894	9	3	5	1
Cellular response to acid chemical	GO:0071229	40/2795	159/16672	0.004430332	0.028921375	0.021590515	40	5	31	4
Natural killer cell mediated cytotoxicity	GO:0042267	17/2795	53/16672	0.004574214	0.029756355	0.022213848	17	0	17	0
Positive regulation of protein polymerization	GO:0032273	29/2795	107/16672	0.004692622	0.030350738	0.02265757	29	5	23	1
Pyrimidine ribonucleoside catabolic process	GO:0046133	6/2795	11/16672	0.004736193	0.030350738	0.02265757	6	1	4	1
Anatomical structure regression	GO:0060033	6/2795	11/16672	0.004736193	0.030350738	0.02265757	6	1	5	0
Osteoblast proliferation	GO:0033687	10/2795	25/16672	0.00491997	0.031241809	0.023322776	10	1	8	1
Regulation of blood pressure	GO:0008217	41/2795	165/16672	0.005039925	0.031858709	0.023783307	41	4	33	4
Zymogen activation	GO:0031638	13/2795	37/16672	0.005316698	0.033195208	0.024781036	13	3	10	0
Collagen metabolic process	GO:0032963	29/2795	108/16672	0.005409722	0.033700697	0.025158396	29	5	23	1
Regulation of mitochondrial depolarization	GO:0051900	8/2795	18/16672	0.005513484	0.033856424	0.02527465	8	1	7	0
Podosome assembly	GO:0071800	8/2795	18/16672	0.005513484	0.033856424	0.02527465	8	3	4	1
Acetylcholine receptor signaling pathway	GO:0095500	8/2795	18/16672	0.005513484	0.033856424	0.02527465	8	4	4	0
Response to acetylcholine	GO:1905144	8/2795	18/16672	0.005513484	0.033856424	0.02527465	8	4	4	0
Cellular response to acetylcholine	GO:1905145	8/2795	18/16672	0.005513484	0.033856424	0.02527465	8	4	4	0
Nitric oxide biosynthetic process	GO:0006809	19/2795	63/16672	0.005984257	0.03626901	0.027075705	19	0	19	0
Receptor metabolic process	GO:0043112	38/2795	152/16672	0.006071061	0.036621444	0.027338805	38	11	26	1
Syncytium formation by plasma membrane fusion	GO:0000768	15/2795	46/16672	0.006334844	0.037794929	0.028214841	15	4	11	0
Cyclic nucleotide catabolic process	GO:0009214	9/2795	22/16672	0.006344246	0.037794929	0.028214841	9	4	4	1
Positive regulation of cardiocyte differentiation	GO:1905209	9/2795	22/16672	0.006344246	0.037794929	0.028214841	9	2	7	0
Regulation of organic acid transport	GO:0032890	7/2795	15/16672	0.006801198	0.039856964	0.029754201	7	2	3	2
Positive regulation of glycogen biosynthetic process	GO:0045725	7/2795	15/16672	0.006801198	0.039856964	0.029754201	7	2	4	1
Cell differentiation involved in metanephros development	GO:0072202	10/2795	26/16672	0.006811632	0.039856964	0.029754201	10	1	9	0
Regulation of alcohol biosynthetic process	GO:1902930	12/2795	34/16672	0.007003623	0.040597765	0.030307227	12	4	7	1
Neuron migration	GO:0001764	34/2795	134/16672	0.007170845	0.041419288	0.030920514	34	8	25	1
Multicellular organismal homeostasis	GO:0048871	72/2795	326/16672	0.007174995	0.041419288	0.030920514	72	12	56	4
Cellular response to external stimulus	GO:0071496	58/2795	254/16672	0.007310818	0.042029679	0.031376186	58	12	43	3
Regulation of circadian sleep/wake cycle, sleep	GO:0045187	8/2795	19/16672	0.008141779	0.045587435	0.034032138	8	3	5	0
Actin filament polymerization	GO:0030041	39/2795	160/16672	0.008546097	0.047285076	0.035299468	39	6	31	2
Negative regulation of homeostatic process	GO:0032845	42/2795	175/16672	0.008651382	0.047770677	0.035661981	42	10	32	0
Response to retinoic acid	GO:0032526	27/2795	102/16672	0.008682974	0.047850552	0.03572161	27	4	22	1
Response to drug	GO:0042493	85/2795	397/16672	0.008716985	0.04795915	0.035802681	85	21	57	7
Membrane protein ectodomain proteolysis	GO:0006509	13/2795	39/16672	0.008745593	0.04795915	0.035802681	13	4	9	0
Alcohol metabolic process	GO:0006066	63/2795	282/16672	0.008809591	0.048215471	0.035994031	63	17	42	4
Calcium-independent cell–cell adhesion via plasma membrane cell-adhesion molecules	GO:0016338	9/2795	23/16672	0.008896008	0.048545794	0.036240625	9	6	3	0
Single-organism behavior	GO:0044708	83/2795	387/16672	0.00905125	0.049296674	0.036801175	83	29	47	7
Single-organism nuclear import	GO:1902593	60/2795	267/16672	0.00915905	0.049622066	0.037044088	60	9	47	4
Ribonucleoside diphosphate metabolic process	GO:0009185	25/2795	93/16672	0.009208122	0.049622066	0.037044088	25	7	14	4
Regulation of natural killer cell mediated cytotoxicity	GO:0042269	11/2795	31/16672	0.009244197	0.049622066	0.037044088	11	0	11	0
Regulation of response to cytokine stimulus	GO:0060759	36/2795	146/16672	0.009298433	0.049865303	0.037225671	36	1	33	2

### Methylation of the mTOR Pathway in SEGAs

**Fig. 2:**
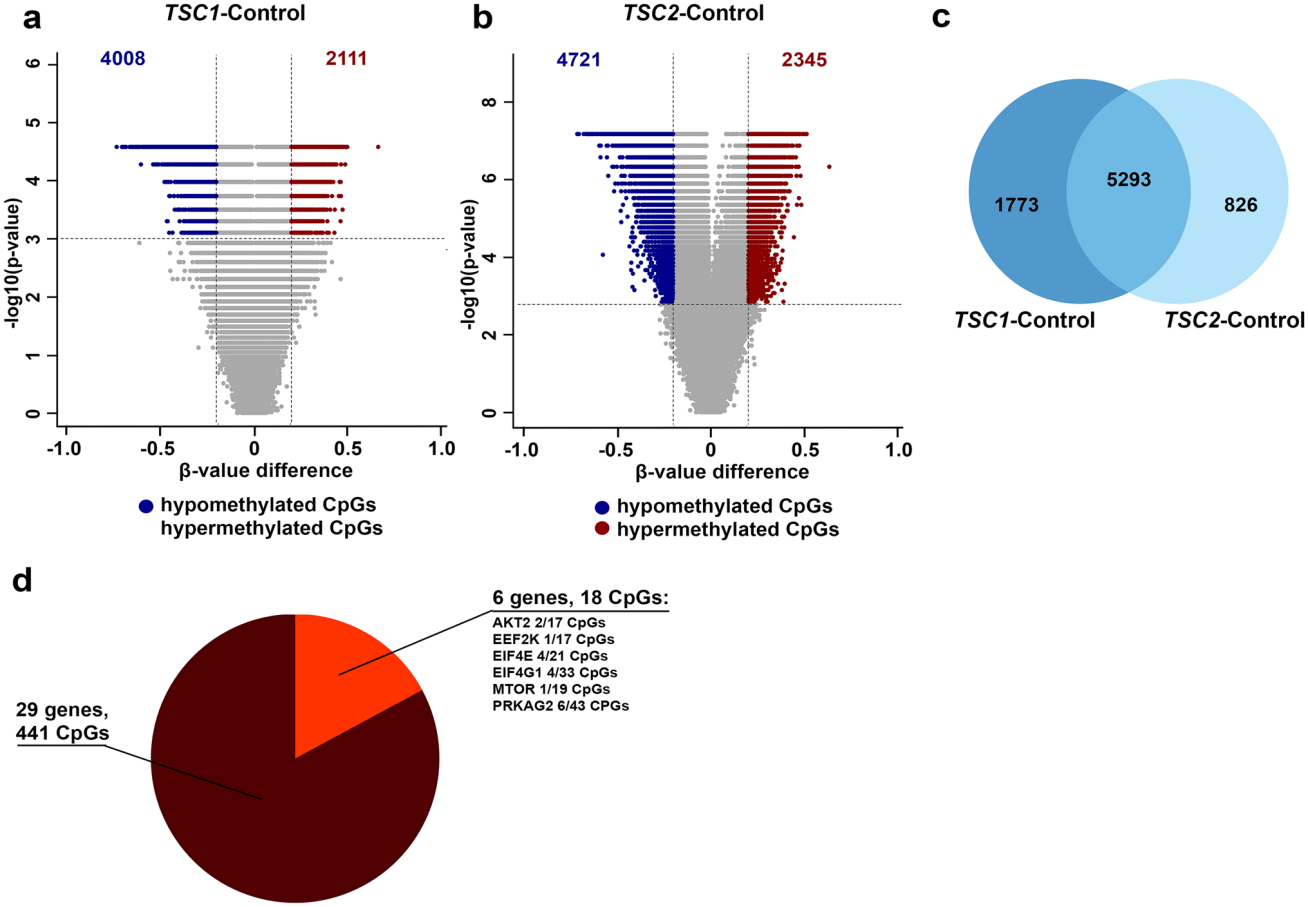
Methylation of the mTOR pathway in SEGAs. **a** Volcano plot showing the differentially methylated CpGs located in the TSS-associated regions (adjusted *p*-value < 0.01 and a *β*-value difference of > 0.2) between *TSC1* mutated SEGAs and control tissue (TSC1-control). A total of 4008 CpGs were hypomethylated and 2111 were hypermethylated in TSC1-control. **b** Volcano plot showing the differentially methylated CpGs on the TSS-associated regions (adjusted *p*-value < 0.01 and a *β*-value difference of > 0.2) between *TSC2* mutated SEGAs and control tissue (TSC2-control). A total of 4008 CpGs were hypomethylated and 2111 were hypermethylated in TSC2-control. **c** Venn diagram showing the overlap of CpGs between TSC1-control and TSC2-control. 5293 CpGs were overlapping, whereas 1773 CpGs were only found differentially methylated in TSC1-control and 826 CpGs in TSC2-control. **d** Pie chart showing the distribution of differentially methylated and not differentially methylated mTOR pathway related genes (based on Reactome). A total of 18 CpGs located on 6 mTOR pathway related genes were differentially methylated, whereas 441 CpGs located on 29 mTOR pathway related genes were not differentially methylated

In order to compare the *TSC1* mutated SEGA samples with the *TSC2* mutated SEGA samples, three differential analyses were carried out: *TSC1* mutated SEGAs compared to control (TSC1-control) and *TSC2* mutated SEGAs compared to control (TSC2-control) and *TSC1* mutated SEGAs compared to *TSC2* mutated SEGAs (TSC1–TSC2). We identified 6119 differentially methylated CpGs in TSC1-control (Fig. 2a), 7066 differentially methylated CpGs in TSC2-control (Fig. 2b) and no CpGs differentially methylated in TSC1–TSC2 (adjusted *p*-value 0.01, *β*-value difference of > 0.2, TSS-associated regions). The majority of the differentially methylated CpGs in TSC1-control and TSC2-control were overlapping (5293 CpGs; Fig. 2c).

We further evaluated the methylation of mTOR pathway related genes by extracting CpGs that were located on genes from the Reactome-based mTOR pathway or mTORC1 signaling pathway. A total of 459 CpGs were located on 35 mTOR pathway related genes of which 18 CpGs located on 6 genes (2/17 CpGs on *AKT2*, 1/17 on *EEF2K*, 4/21 on *EIF4E*, 4/33 on *EIF4G1*, 1/19 on *MTOR* and 6/43 on *PRKAG2*) were differentially methylated (Fig. 2d). The majority of these CpGs were hypomethylated (15/18), whereas 3 CpGs located on *PRKAG2* were hypermethylated.

### Expression of Inflammation, mTOR Activation, Glial and Neuronal Markers in SEGAs

**Fig. 3:**
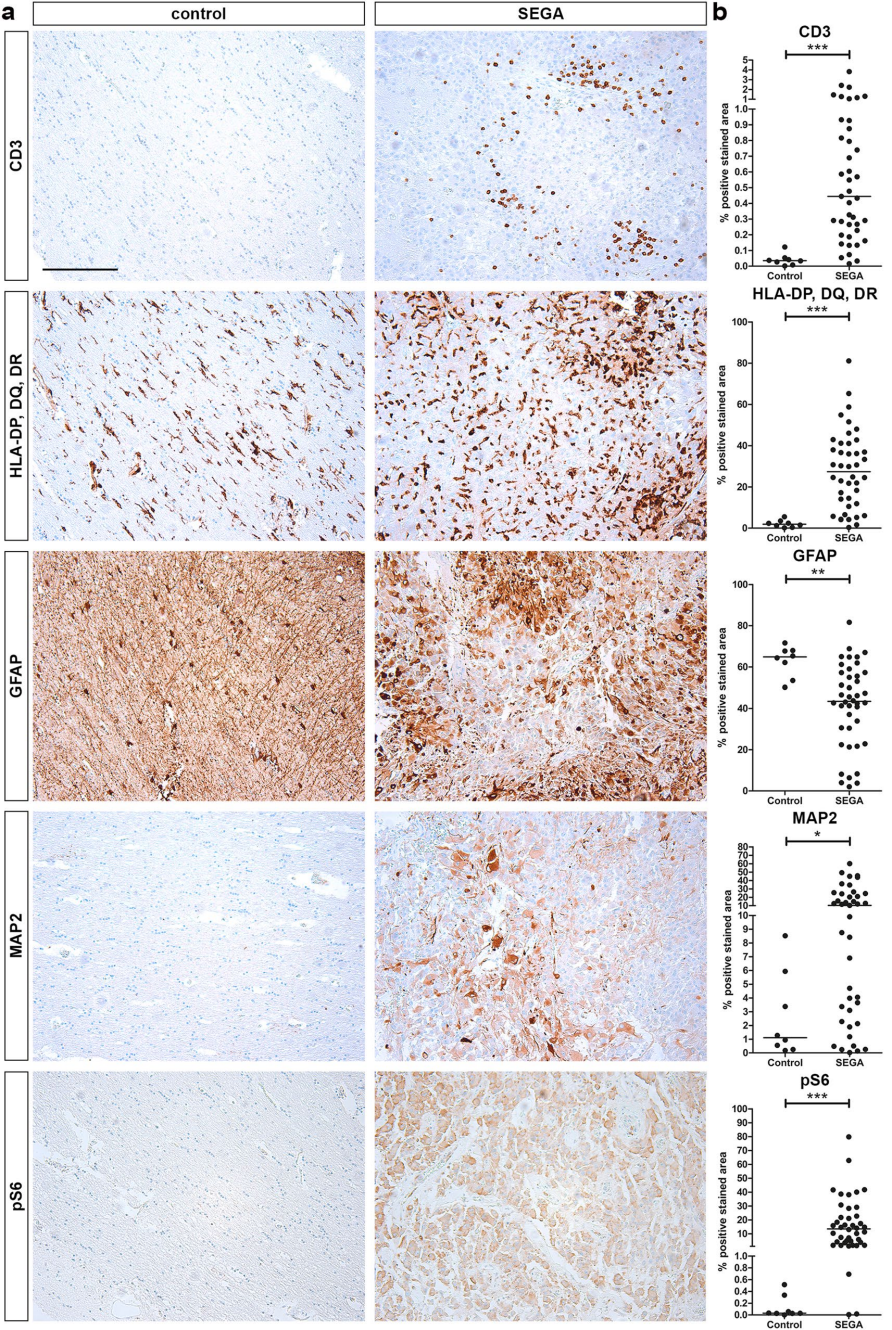
Expression of inflammatory, mTOR activation, glial and neuronal markers SEGAs. **a** Immunohistochemistry showing the expression of CD3, HLA-DP/DQ/DR, pS6, GFAP and MAP2 in SEGA and periventricular control. Scale bar = 200 μm. **b** Quantification of immunohistochemistry indicated in % of positive area of CD3, HLA-DP/DQ/DR, pS6, GFAP and MAP2 in SEGA and periventricular control. An higher expression of CD3, HLA-DP/DQ/DR, pS6 and MAP2 and a lower expression of GFAP was found in SEGA compared to control. *p-value < 0.05, **p-value < 0.01, ***p-value < 0.001, Mann–Whitney *U* test

SEGAs are considered mixed glio-neuronal tumors, with mTOR activity and presence of inflammation markers. Therefore, we wanted to evaluate the commonalities and differences in the expression of CD3, HLA-DP/DQ/DR, GFAP, MAP2 and pS6 in 42 SEGAs and 8 location-matched controls. In periventricular control tissue CD3, MAP2 and pS6 were not detected, whereas a moderate expression of HLA-DP/DQ/DR and high expression of GFAP was seen (Fig. 3a). In SEGA, we found several positive CD3 cells and observed an overall increase in positive area for CD3 in SEGA compared to control tissue (Fig. 3b; *p* < 0.0001). HLA-DP/DQ/DR, GFAP, MAP2 and pS6 were expressed in a heterogeneous manner in SEGAs (Fig. 3a). The percentage of positive area of HLA-DP/DQ/DR (*p* < 0.0001), MAP2 (*p* = 0.0114) and PS6 (*p* < 0.0001) were increased in SEGA compared to control tissue, whereas the positive area for GFAP was decreased in SEGA (*p* = 0.0016).

Spearmans rank correlation revealed a weak positive correlation between the expression of CD3 and HLA-DP/DQ/DR (*r* = 0.347; *p* = 0.026), pS6 and HLA-DP/DQ/DR (*r* = 0.368; *p* = 0.016), and GFAP and HLA-DP/DQ/DR (*r* = 0.325; *p* = 0.036) in SEGA. Spearmans rank correlation with clinical data revealed a weak positive correlation between age at surgery and CD3 (*r* = 0.3197; *p* = 0.0416) and a negative correlation between tumor size and CD3 (*r* =  − 0.4331; *p* = 0.0118), HLA-DP/DQ/DR (*r* =  − 0.4370; *p* = 0.0098), MAP2 (*r* =  − 0.4746; *p* = 0.0046) and pS6 (*r* =  − 0.4884; *p* = 0.0034).

### Two Distinct Methylation Groups in SEGAs

**Fig. 4:**
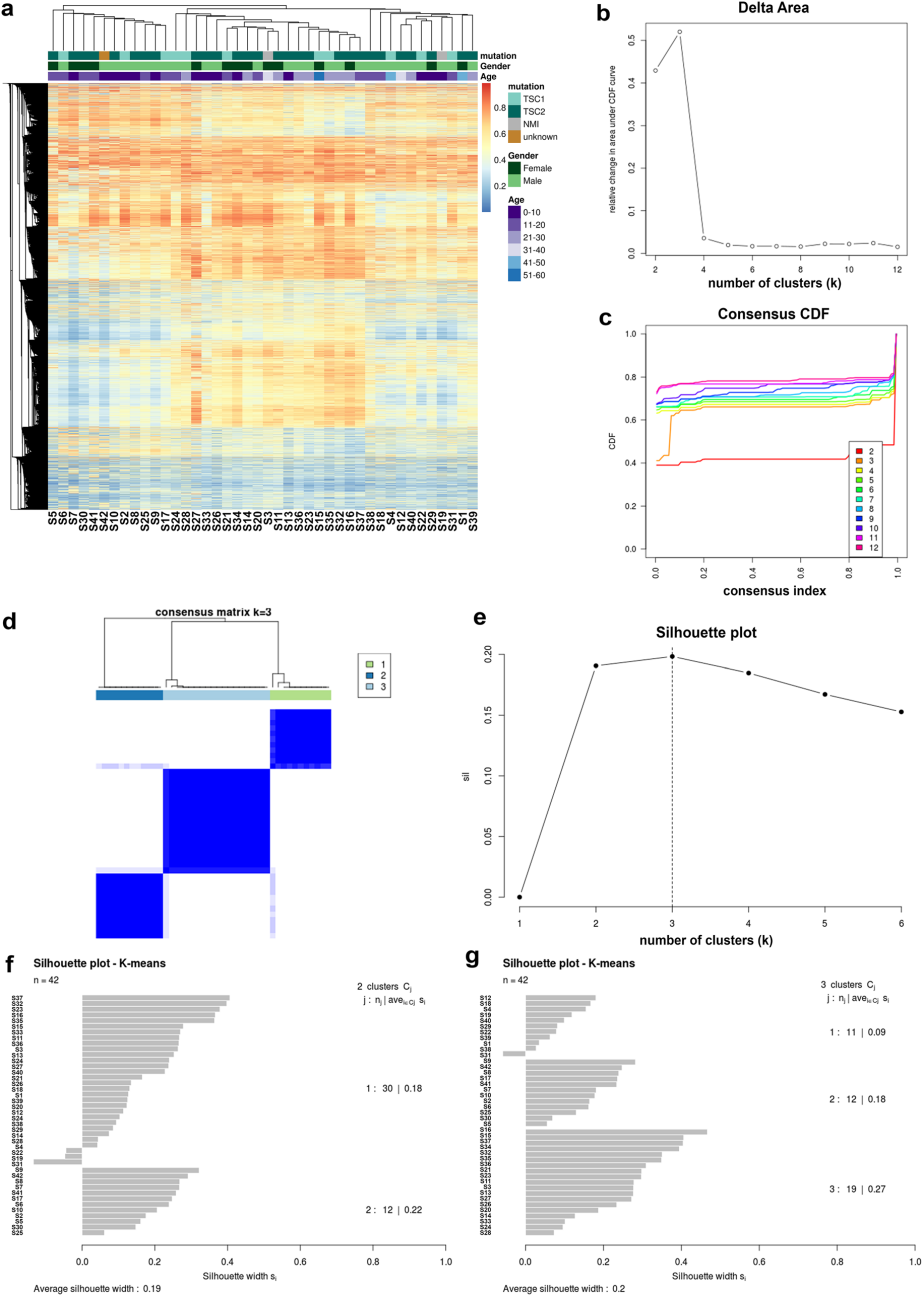
Two robust groups found in the methylation data of SEGAs. **a** Heatmap using hierarchical clustering showing two/three subgroups in SEGA. **b**–**d** Consensus clustering showing that *k* = 3 is most robust indicated by delta area plot (**b**) consensus Cumulative Distribution Function (CDF) plot (**c**) and consensus matrix for *k* = 3 (**d**). **e** Silhouette plot showing the average silhouette with for each k, indicating that *k* = 3 is most robust. **f** Barchart of the silhouette clustering with for *k* = 2 showing which samples cluster together. **g** Barchart of the silhouette clustering with for *k* = 3 showing which samples cluster together

To evaluate potential subgroups within the SEGA samples the top 5% most variable CpGs were analysed with hierarchical clustering, consensus clustering and silhouette clustering. Hierarchical clustering indicated 2 major groups with one group subdividing into two smaller groups (Fig. 4a). This was confirmed by both consensus clustering (Fig. 4c–d) and silhouette plots (Fig. 4e–g), which indicated *k* = 3 as the most robust number of clusters. To assess other potential confounders on the methylation profile another PVCA was performed, showing that the major variance between the SEGA samples matched with the identified subgroups *k* = 3, followed by the subgroups *k* = 2 (Supplementary Fig. 1b). Other clinical data contributed minimally to the overall variance seen amongst the samples. Clustering of subgroups was re-evaluated in an additional independent SEGA cohort from Heidelberg (50 additional cases) showing the robustness of the two groups (Supplementary Fig. 3).

**Fig. 5:**
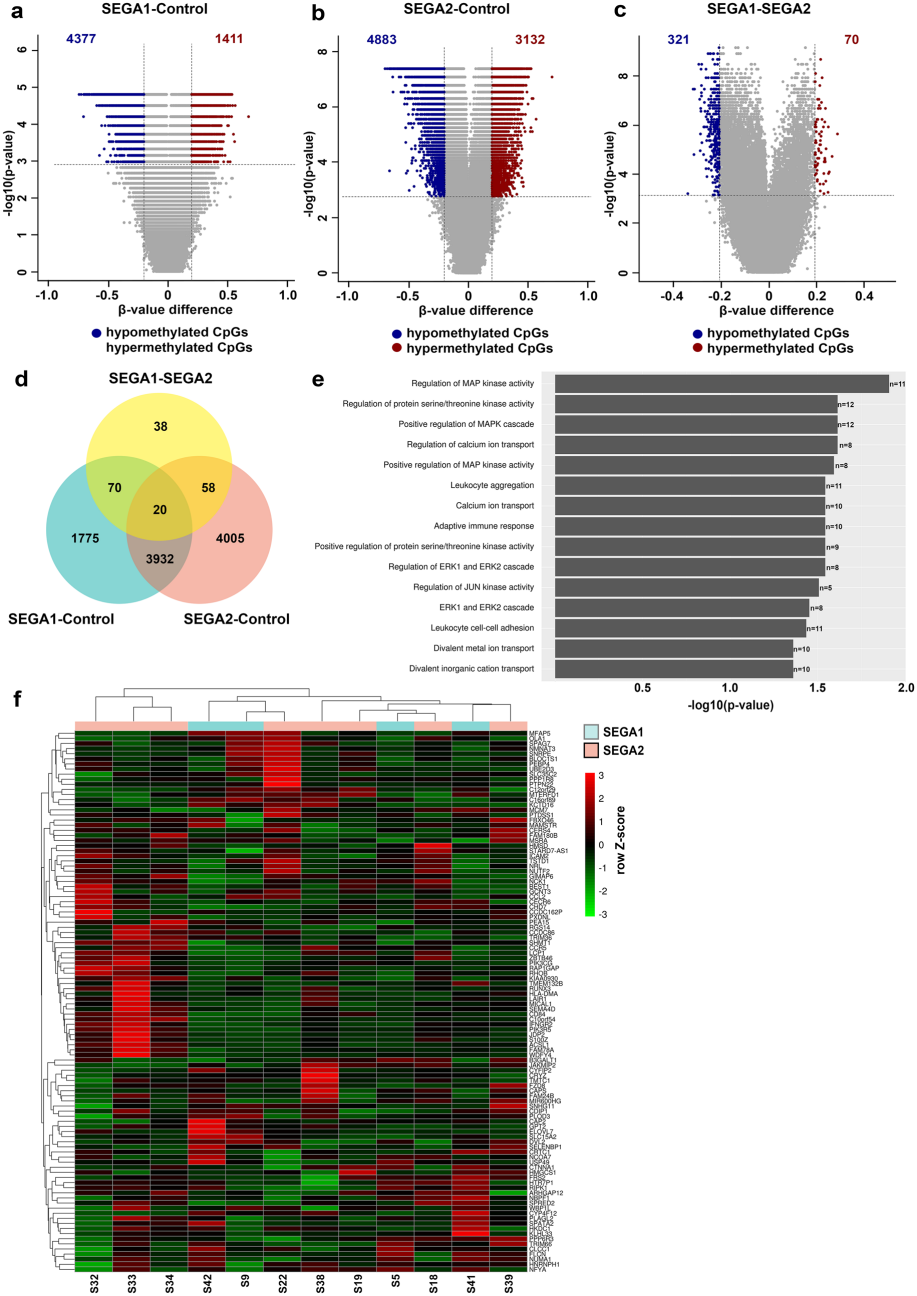
Unique gene expression between two SEGA subgroups. **a** Volcano plot showing the differentially methylated CpGs on the TSS-associated regions (adjusted *p*-value < 0.01 and a *β*-value difference of > 0.2) between one subgroup of SEGAs and control tissue (SEGA1–control). A total of 4377 CpGs were hypomethylated and 1411 were hypermethylated in SEGA1-control. **b** Volcano plot showing the differentially methylated CpGs on the TSS-associated regions (adjusted *p*-value < 0.01 and a *β*-value difference of > 0.2) between the other subgroup of SEGAs and control tissue (SEGA2-control). A total of 4883 CpGs were hypomethylated and 3132 were hypermethylated in SEGA2-control. **c** Volcano plot showing the differentially methylated CpGs on the TSS-associated regions (adjusted *p*-value < 0.01 and a *β*-value difference of > 0.2) between the two subgroups of SEGA (SEGA1–SEGA2). A total of 321 CpGs were hypomethylated and 70 were hypermethylated in SEGA1–SEGA2. **d** Venn diagram showing the overlap of genes corresponding to the differentially methylated CpGs between SEGA1-control, SEGA2-control and SEGA1-SEGA2. Genes overlapping between SEGA1-control and SEGA1-SEGA2 but not with SEGA2-control were considered unique for SEGA1. Genes overlapping between SEGA2-control and SEGA1-SEGA2 but not with SEGA1-control were considered unique for SEGA2 (70 genes SEGA1 unique and 58 genes SEGA2 unique). **e** Bar chart showing the GO terms enriched for the SEGA1 and SEGA2 unique genes. Each GO term is listed on the y-axis, the log10(1/adjusted p-value) on the *x*-axis and the n is equal to the number of differentially methylated genes in each GO term. **f** Heatmap with *Z*-score hierarchical clustering for the RNA expression data of 12 SEGAs. Each row indicates one of unique SEGA1 or SEGA2 genes based on the methylation data that was expressed on RNA level in SEGA. The color scale means the gene expression standard deviations from the mean, with green for low expression and red for the high expression levels

We further investigated the two largest groups identified and performed differential testing between group 1 compared to control (SEGA1-control), group 2 compared to control (SEGA2-control) and group 1 compared to group 2 (SEGA1–SEGA2). We found 4377 hypomethylated and 1411 hypermethylated CpGs in SEGA1-control (Fig. 5a), 4883 hypomethylated and 3132 hypermethylated CpGs in SEGA2-control (Fig. 5b) and 321 hypomethylated and 70 hypermethylated CpGs in SEGA1-SEGA2 (Fig. 5c; adjusted *p*-value 0.01, *β*-value difference of > 0.2, TSS-associated regions). In order to identify differentially methylated genes, genes corresponding to the differentially methylated CpGs were extracted. Genes that were overlapping between SEGA1-control and SEGA1–SEGA2 and did not overlap with SEGA2-control were considered unique for SEGA1, whereas genes that overlapped between SEGA2-control and SEGA1–SEGA2 but did not overlap with SEGA1-control were considered unique for SEGA2 (70 SEGA1 unique genes and 58 SEGA2 unique genes; Fig. 5d). GO analysis revealed 15 GO terms enriched for these 128 unique genes, which were related mainly to the MAPK cascade and adaptive immune response (*p*-adjusted < 0.05; Fig. 5e). We further evaluated the RNA expression of these 128 genes and performed correlations between the normalized count matrix of genes that were expressed (106/128 genes) and the β-values of the corresponding CpGs. We identified 11 genes that inversely correlated with their corresponding CpG (Table 3). Based on the RNA expression no clear clustering was found between SEGA1 and SEGA2, although the number of cases in each group was relatively small (Fig. 5f).

**Table 3:** Correlations between SEGA1 or SEGA2 unique genes and their corresponding CpGs

Gene-CpG	*p*-value	*R*
*MFAP5*-cg07708516	0.000530223	− 0.824175824
*MFAP5*-cg15815843	0.000620988	− 0.818681319
*MFAP5*-cg18574995	0.00145419	− 0.785714286
*SLC15A2*-cg10523671	0.025167117	− 0.615384615
*GPT2*-cg05380921	0.038992623	− 0.576923077
*CLCC1*-cg05048348	0.034586058	− 0.587912088
*CYP4F12*-cg04608829	0.007203143	− 0.704264765
*KLHL33*-cg10982443	0.019586595	− 0.635488909
*C12orf29*-cg02981078	0.005023238	− 0.725274725
*SELENBP1*-cg24486037	0.00731857	− 0.703296703
*MTERFD1*-Cg16177163_1	0.03252444	− 0.593406593
*ICAM2*-cg24080793	0.025167117	− 0.615384615
*CHD7*-cg20078807	0.020516043	− 0.631868132

**Fig. 6:**
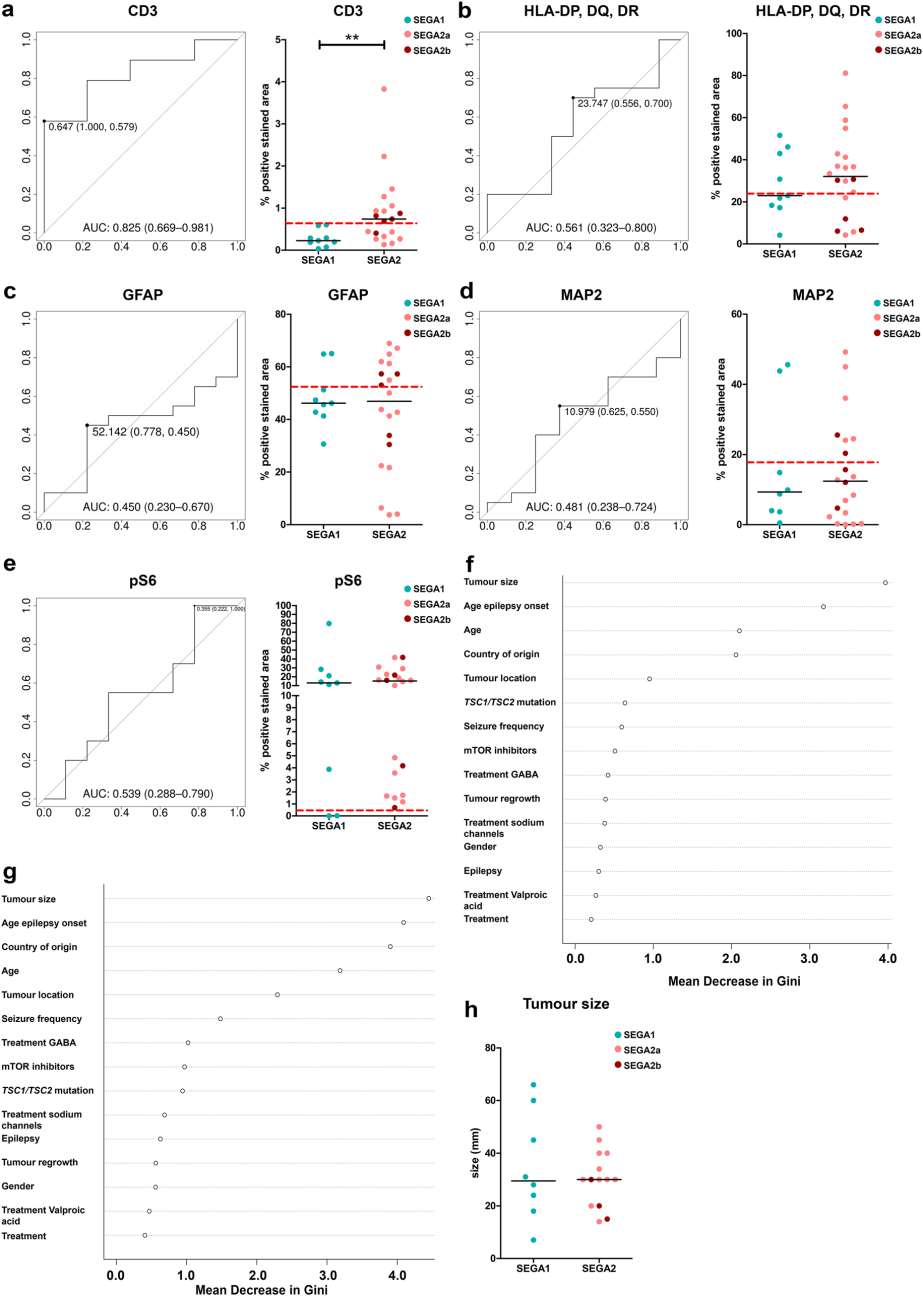
Detection of SEGA subgroups based on histological markers and clinical data. **a**–**e** ROC curves and scatterplots for detection of SEGA1 or SEGA2 based on CD3 (**a**), HLA-DP/DQ/DR (**b**), GFAP (**c**), MAP2 (**d**) and pS6 (**e**) positivity. The point in the ROC curve indicates the most optimal % of positivity to separate the SEGA1 and SEGA2 group, followed by the proportion of SEGA1 and SEGA2 that is correctly detected. Scatterplots show the spread between samples for the % of positive area for SEGA1 (blue) and SEGA2 (SEGA2a light red and SEGA2b dark red). The red line indicates the most optimal % of positivity to separate the SEGA1 and SEGA2 group based on the ROC curve. *p-value < 0.05, **p-value < 0.01, ***p-value < 0.001, Mann–Whitney *U* test. **f** Variable Importance plots obtained from Random Forest in R for detection of SEGA1 and SEGA2 based on the clinical data (Table 1). Each point represents the mean decrease Gini value, indicative of the importance of each variable. Variables are listed from most important to least important. Antiepileptic drugs were also categorized in 3 groups: GABA blockers (treatment GABA), valproic acid (treatment valproic acid) and sodium channel blockers (treatment sodium channels).** g** Variable Importance plots obtained from Random Forest in R for detection of SEGA1, SEGA2a and SEGA2b based on the clinical data (Table 1). Each point represents the mean decrease Gini value, indicative of the importance of each variable. Variables are listed from most important to least important. Antiepileptic drugs were also categorized in 3 groups: GABA blockers (treatment GABA), valproic acid (treatment valproic acid) and sodium channel blockers (treatment sodium channels). **h** Scatterplot showing no difference in tumor size (mm) in SEGA1 (blue) compared to SEGA2 (SEGA2a light red and SEGA2b dark red)

Next, we investigated if the expression of CD3, HLA-DP/DQ/DR, GFAP, MAP2 or pS6 could explain the subgroups found in the methylation data. We found a higher positive area of CD3 in SEGA2 compared to SEGA1 (*p* = 0.0068; Fig. 6a boxplot). No difference was found between SEGA1 and SEGA2 for the other markers (Fig. 6b–e; boxplots) or between the subgroups of SEGA2, SEGA2a and SEGA2b (Fig. 6a–e; boxplots). Using a ROC analysis, we identified CD3 positive area as the best predictor for dividing SEGA1 from SEGA2 (Fig. 6a; AUC = 0.825) compared to HLA-DP/DQ/DR (Fig. 6b; AUC = 0.561), GFAP (Fig. 6c; AUC = 0.450), MAP2 (Fig. 6d; AUC = 0.481) and pS6 (Fig. 6e; AUC = 0.539). Furthermore, using Random Forest we found that none of the clinical data could properly separate between SEGA1 and SEGA2 (Fig. 6f) or between SEGA1, SEGA2a and SEGA2b (Fig. 6g). The tumor size contributed most to separating the two groups but showed no significant difference between SEGA1 and SEGA2 (Fig. 6h).

## Discussion

In this study, we performed DNA methylation profiling of SEGAs from TSC patients and showed that the differential methylation profile between SEGAs and control tissue was enriched for GO terms including the adaptive immune system, T cell activation, leukocyte mediated immunity, extracellular structure organization and the ERK1 & ERK2 cascade. Histological markers for T cells, microglia reactivity, mTOR activation and neurons were higher expressed, whereas the glial marker GFAP was lower expressed in SEGA compared to periventricular control tissue. Furthermore, we identified two robust subgroups in the DNA methylation of SEGA, with a distinct methylation profile of genes related to the adaptive immune response and the MAPK pathway. Moreover, we found differences in positivity of the T cell marker CD3 between the two largest subgroups.

Previous studies on SEGA methylation have shown that SEGAs are a unique entity among CNS tumors (Martin et al. 2017; Capper et al. 2018a,b). However, the molecular mechanisms targeted by methylation changes in SEGA have not been well studied. In the present study, we identified substantial methylation changes in SEGAs compared to periventricular control tissue that appeared to be independent of the *TSC1*/*TSC2* mutation or other clinical information available. GO analysis showed an enrichment of the adaptive immune system, T cell activation, leukocyte-mediated immunity, extracellular structure organization and the ERK1 & ERK2 cascade. Previous gene expression studies on SEGA found differential expression of similar pathways, indicating that these pathways are already affected on DNA level and might therefore be important drivers in SEGA pathogenesis (Bongaarts et al. 2019; Martin et al. 2017; Tyburczy et al. 2010). Differential expression of genes related to the immune system and the ECM organization has also been seen in cortical tubers (Martin et al. 2017; Mills et al. 2017). Furthermore, several studies have documented dysregulation of inflammation and ECM organization related pathways in cortical tubers, suggesting that these processes might be conserved across TSC pathology (Boer et al. 2008, 2010; Prabowo et al. 2013; Broekaart et al. 2019). Therefore, it would be of interest to investigate if DNA methylation changes related to these processes are also present in cortical tubers and other TSC lesions.

The role of mTOR pathway activation due to loss of function mutations in *TSC1*/*TSC2* in TSC is well established. Furthermore, loss of heterozygosity (LOH) of *TSC1* or *TSC2* has been reported in approximately 80% of SEGAs and has also been found in other TSC hamartomas (Martin et al. 2017; Bongaarts et al. 2017; Chan et al. 2004). In vitro experiments show that mTOR inhibitors can reduce cell size and cell proliferation of SEGA cells. Currently, mTOR inhibitors are amongst the treatment options for SEGA associated with TSC (Franz et al. 2015). Therefore, we aimed to investigate methylation changes on genes related to the mTOR signaling pathway. GO term analysis on the differentially methylated CpGs between SEGA and control tissue did not reveal the mTOR pathway as a principal target. Moreover, by directly investigating mTOR pathway related genes, we found only a small number of differentially methylated CpGs on 6/35 mTOR related genes, indicating that DNA methylation changes most likely do not contribute to the mTOR activation in SEGA. It has been suggested that *TSC1*/*TSC2* epigenetic silencing might contribute to tumor formation in TSC and could explain cases where the second hit mutation in *TSC1*/*TSC2* is not found (Jiang et al. 2005; Lesma et al. 2009). In accordance with previous study, we did not find evidence of epigenetic silencing of the promoter of *TSC1* or *TSC2* in SEGA (Martin et al. 2017).

Initial studies suggested an astrocytic nature of SEGAs, whereas more recent studies demonstrate a mixed glio-neuronal phenotype, with mTOR activity and presence of inflammation markers (Bongaarts et al. 2017; Boer et al. 2008; Chan et al. 2004; Buccoliero et al. 2009, 2016). We evaluated the expression of CD3, HLA-DP/DQ/DR, GFAP, MAP2 and pS6 using whole slide scanning in 42 SEGAs and 8 location and age matched controls. In accordance with previous literature, we confirmed the presence of inflammation markers and mTOR pathway activation in SEGA compared to control tissue (Bongaarts et al. 2017; Boer et al. 2008; Chan et al. 2004; Buccoliero et al. 2009, 2016). Previous research showed that the mTOR activation is mainly present in giant cells and not in spindle cells of SEGA, which could explain the variability between the SEGA samples seen in this study (Buccoliero et al. 2016). The mTOR pathway can also regulate inflammatory responses and a previous study has shown that HLA-DR positive microglial cells were localized around giant cells in SEGA (Boer et al. 2008; Lim et al. 2003). In accordance, we found a positive correlation between the expression HLA-DP/DQ/DR and pS6. Furthermore, we found expression of both GFAP and MAP2 in SEGAs confirming a glio-neuronal nature of SEGAs. However, the percentage of positive GFAP area was lower in SEGA compared to control tissue. This lower expression could be explained by the diffuse staining of GFAP, which has been reported in prior immunohistochemical studies, indicating that some SEGA cells might lose their glial phenotype (Buccoliero et al. 2009, 2016). It could be that GFAP negative SEGA cells have a more neuronal expression, however, no negative correlation was found between the expression of GFAP and MAP2 in SEGA.

Interestingly, we identified two subgroups (SEGA1 and SEGA2) in the SEGA methylation data with one group subdividing further into two smaller groups. These two groups were distinct in the methylation of genes related to the adaptive immune response and the MAPK cascade. However, no correlation was found between the methylation and RNA expression of these specific genes. Due to the complexity of RNA expression regulation, the effect of methylation changes might not be directly reflected in the RNA expression data. Higher expression of the T cell marker CD3 was found in SEGA2 compared to SEGA1 confirming differences in the adaptive immune response between the two groups. It could be possible that these methylation subgroups reflect differences in inflammatory cell content. However, it must be noted that the differences are small and only detectible with quantification. Furthermore, CD3 was not found differentially methylated between SEGA1 and SEGA2, suggesting an indirect effect of methylation on CD3 expression. The precise role of T-cells in SEGAs is still unknown, it could indicate a more responsive immune response to tumor cells but we also know that, neuroinflammation can increase the expression and activity of MMPs, which are increased in SEGAs and can play a role in tumorgenesis (Bongaarts et al. 2020). The MAPK pathway has been shown to be an important pathway for SEGA growth and has been suggested as a novel target for therapy for patients with SEGA (Bongaarts et al. 2019; Tyburczy et al. 2010; Mi et al. 2009). Therefore, the differences in methylation of this pathway between the two groups found in this study is interesting and could potentially reflect how these tumors would respond to MAPK inhibitors. Since MAPK inhibitors are not routinely used for SEGA treatment this could not be evaluated within this study. Furthermore, none of the clinical data available could explain the subgroups found, including the TSC mutation. We did not find any confounding effects, however we cannot exclude confounding by other parameters such as Body Mass Index (BMI). It is very well possible that these two groups do reflect other important clinical features that were not evaluated in this study, such as tumor progression, or any differences in the clinical phenotypes either related or unrelated to the TSC mutation. Moreover, since the majority of patients included were not treated with mTOR inhibitors, it cannot be excluded that the biological make up of these subgroups reflect potential response to mTOR inhibitors. Therefore, DNA methylation analysis on SEGAs in retrospective studies are highly needed in order to unravel the clinical relevance of these subgroups.

## Conclusions

Overall, this study shows that the DNA methylation profile of SEGAs is enriched for the immune system and the MAPK pathway and the ECM organization, strengthening the importance of these pathways in SEGA development and suggests that therapeutic intervention on DNA level could be useful. Moreover, we identified two subgroups in SEGA that seem to be driven by changes in the adaptive immune response and MAPK pathway. Although the clinical relevance of these subgroups remains uncertain they could potentially reflect tumor progression or response to treatment other than anti-epileptic drugs and deserve further investigation.

## Materials and Methods

### SEGA Tumor Specimens

A total of 55 SEGA specimens were obtained from the following sites: the Amsterdam University Medical Center (location AMC), the University Medical Center Utrecht, University Medical Center Groningen, Medical University of Vienna, Children’s Memorial Health Institute in Warsaw, Meyer Children's Hospital in Florence, University Hospital Erlangen, University Hospital Münster, Hacettepe University in Ankara, the University Hospital of Santa Maria (CHLUN) in Lisbon, and the North Bristol NHS Trust as part of the UK Brain Archive Information Network (BRAIN UK: Ref.: 16/002). Fifty-one of the SEGA samples were obtained from patients that met the clinical diagnostic criteria for TSC. Histological diagnosis was confirmed following the current WHO classification guidelines by two independent neuropathologists (Louis et al. 2016). *TSC1*/*TSC2* mutation analysis was performed in blood or tumor sample DNA (for samples with a sufficient amount of DNA) as part of routine clinical care or was detected using massively parallel sequencing as described previously (Table 1) (Bongaarts et al. 2017). The following clinical data was collected from medical records: age at time of surgery, *TSC1*/*TSC2* mutation status, gender, localization of the SEGA, size of the tumor, age at seizure onset, seizure frequency, drug management at time of surgery (including treatment with mTORC1 inhibitors), tumor recurrence/regrowth and presence of other TSC related malformations. The ethnicity of all patients was Caucasian. Periventricular brain tissue was used as control and was obtained from autopsy controls without a history of TSC, epilepsy, brain tumors or other neurological manifestations. Specimens were obtained and used in accordance with the Declaration of Helsinki and this study was approved by the Medical Ethics Committees of each institution.

### DNA Extraction & 450k Methylation Analysis

DNA was extracted from FFPE SEGA tumor samples (*n* = 42) and location matched controls (*n* = 8). Representative tumor regions were identified on hematoxylin & eosin sections for cases in which hemorrhages were prominent and were macrodisected from unstained sections (10-μm-thick) to reach a tumor cell content of 70% or more. For controls a punch of 2 mm diameter and up to 3 mm depth was taken from the periventricular zone. DNA was extracted using BiOstic FFPE Tissue DNA Isolation kit (MO BIO) according to the manufacturer's instructions and DNA quality was evaluated. 200–300 ng of DNA per sample was analysed using Illumina Infinium HumanMethylation450 BeadChip (450k) arrays according to the manufacturer’s instructions at the Genomics and Proteomics Core Facility of the DKFZ (Heidelberg, Germany).

### RNA Isolation and RNA Sequencing

Frozen tissue of 12 SEGAs was homogenized with Qiazol Lysis Reagent (Qiagen Benelux, Venlo, The Netherlands) and total RNA was isolated using the miRNeasy Mini kit (Qiagen Benelux, Venlo, the Netherlands) according to the manufacturer’s instructions. RNA concentration was determined using a Qubit® 2.0 Fluorometer (Life Technologies, Carlsbad, CA, USA) and the RNA integrity was assessed using a Bioanalyser 2100 (Agilent). Library preparation and sequencing were completed at GenomeScan (Leiden, the Netherlands). The Illumina (San Diego, California, USA) NEBNext Ultra Directional RNA Library preparation kit was used to prepare sequencing libraries in accordance to manufacturers guidelines. Clustering and DNA sequencing was performed using the Illumina cBot and HiSeq 4000 according to manufacturer’s protocols. Each library was subjected to paired-end sequencing, producing reads of 150 nucleotides in length with a read-depth of 36 million reads.

Data were processed as previously described (Bongaarts et al. 2019). Briefly, quality control of the reads was done using FastQC v0.11.5 (Babraham Institute, Babraham, Cambridgeshire, UK). Sequence reads were trimmed and filtered using FastQC v0.11.5 (Babraham Institute, Babraham, Cambridgeshire, UK) and Trimmomatic v0.36 (Bolger et al. 2014). Paired-end reads were aligned to the human reference genome (GRCh38) with TopHat2 v2.0.13 and default settings (Kim et al. 2013). The number of reads that mapped to each gene, based on Gencode v25, was determined using featureCounts from the SubRead package (Liao et al. 2014). The count matrix was normalized using the R package DESeq2 (Love et al. 2014). Confounding variables were assessed with PCA and linear regression.

### Bioinformatic Analysis

Raw IDAT files from the 450k were passed to the minfi package in R and quality control was performed using both minfi and shinymethyl. Two samples failed quality control based on the minfi quality control plot and were therefore not included in this manuscript. Normalization included a Noob background correction and dye-correction based on the control probes using the function preprocessFunnorm from the R package minfi, which removes any between-array variation (Fortin et al. 2014). Probes with detection *p*-values of more than 0.01, located on the sex chromosomes, or in SNPs were removed as well as cross-hybridization probes. After these steps, beta (*β*)-values ranging from 0.0 to 1.0 from 421,352 probes were used for further analysis.

Using the ConsensusClusterPlus package (Wilkerson and HaYes 2010), consensus clustering was performed with h-clust average linkage to detect robust clusters, where the metric was 1 minus the Spearmans correlation coefficient. The procedure was run over 1000 iterations and with a sub-sampling ratio of 0.99. Additionally, we applied a silhouette analysis to identify robust clusters. PCA was performed considering all CpG probes. Hierarchical clustering was performed on the top 5% most variable CpG probes using h-clust with average linkage. PCA, PVCA, linear regression, receiver operating characteristic (ROC) analysis and Random Forest (using the R package Random Forest with standard settings) were used to assess potential confounding factors as well as the contribution of histological and clinical data to the clusters identified. To determine the CpGs that were differentially methylated between groups (e.g. SEGA compared to control) a non-parametric Mann–Whitney U test was used at each CpG probe. The distribution of CpGs on the gene (TSS200, TSS1500, 5’UTR and Exon 1, Intergenic region (IGR), 3’UTR or gene body) was evaluated by calculating the percentage of CpGs per gene region. CpGs that were located on multiple genes or transcript variants were counted as one for each corresponding gene region. CpG probes located at the TSS-associated regions (TSS200, TSS1500, 5’UTR and Exon 1) with a benjamini–Hochberg adjusted *p*-value < 0.01 and a *β*-value difference of > 0.2 were considered differentially methylated. Gene ontology (GO) analysis was performed using the R package clusterProfiler (Yu et al. 2012) and the R package missMethyl. In order to identify mTOR pathway methylation changes the mTOR pathway or mTORC1 signaling pathway genelist from the Reactome database was extracted (Croft et al. 2011). CpGs mapping to these genes with a Benjamini–Hochberg adjusted *p*-value < 0.01 and a *β*-value difference of > 0.2 were considered differentially methylated. Clustering of subgroups was re-evaluated in an additional independent SEGA cohort from Heidelberg (50 additional cases) using hierarchical clustering and t-distributed stochastic neighbor embedding (TSNE) plot (Supplementary Fig. 3).

### Immunohistochemistry

Immunohistochemical staining was performed on 42 SEGAs and 8 controls with a Ventana semiautomated staining machine (Benchmark ULTRA; Ventana, Illkirch, France) and the Ventana DAB staining system according to the manufacturer’s protocol. The following antibodies have been used: glial fibrillary acidic protein (GFAP; polyclonal rabbit, DAKO, Glostrup, Denmark; 1:4000; monoclonal mouse; DAKO; 1:50), microtubule-associated protein (MAP2; mouse clone HM2; Sigma 1:100), human leukocyte antigen class II (HLA-DP, DQ, DR; mouse clone CR3/43; DAKO; 1:100), cluster of differentiation 3 (CD3; mouse monoclonal, clone F7.2.38; DAKO; 1:200; T-lymphocytes), phospho-S6 ribosomal protein (pS6 Ser235/236; rabbit polyclonal, Cell Signaling Technology, Beverly, MA, USA; 1:50).

### Evaluation of Immunohistochemistry

Olympus dotSlide system (vs 2.5, Olympus, Tokyo, Japan) was used for whole slide scanning at a × 20 magnification with a resolution of 0.32 μm/pixel. The scans were converted into TIFF files. The DAB positive area was separated from background using an adjusted protocol for segmentation and was corrected for total area of the tissue. For samples with blood contamination, regions of interest (ROI) were selected in order to select representative tumor regions. The overall percentage of positivity was assessed for each case and used for statistical analysis.

### Statistical Analysis

Statistical analysis was performed with GraphPad Prism software (Graphpad software Inc., La Jolla, CA) using the nonparametric Mann–Whitney *U*-test or, for multiple groups, the non-parametric Kruskal–Wallis test followed by Mann–Whitney *U*-test. Correlations were assessed with R using the Spearman’s rank correlation test. An adjusted *p*-value < 0.05 was considered statistically significant except for the differentially methylation analysis where an adjusted *p*-value < 0.01 was considered statistically significant.

## Supplementary Information

Below is the link to the electronic supplementary material.

Supplementary Figure 1. Principle variance component analysis (PVCA) of all the methylation profiles of both SEGA and control samples. **a.** A PVCA was performed to quantify the contribution of various variables to the overall variance between the samples (SEGA n=42 and control tissue n=8). The major contributor to the overall variance was the diagnosis of the samples (SEGA or control). Other clinical data including age at surgery, *TSC1/TSC2* mutation status, gender, localization of the SEGA, size of the tumor, epilepsy, age at seizure onset, seizure frequency, drug management at time of surgery (including treatment with mTORC1 inhibitors), tumor recurrence/regrowth and presence of other TSC related malformations as well as their various interactions contributed minimally to the overall variance seen amongst the samples. The residual is all variance that cannot be explained by the known factors. **b.** A PVCA was performed to quantify the contribution of various variables to the overall variance between the SEGA samples (SEGA n=42). The major contributor to the overall variance of the SEGA samples was the three subgroups (SEGA1, SEGA2a, SEGA2b) found based on the methylation profile, followed by the two subgroups (SEGA1, SEGA2). Other clinical data including age at surgery, *TSC1/TSC2* mutation status, gender, localization of the SEGA, size of the tumor, epilepsy, age at seizure onset, seizure frequency, drug management at time of surgery (including treatment with mTORC1 inhibitors), tumor recurrence/regrowth and presence of other TSC related malformations as well as their various interactions contributed minimally to the overall variance seen amongst the samples. The residual is all variance that cannot be explained by the known factors. (TIF 6492 kb)

Supplementary Figure 2. Principle component analysis (PCA) of all the methylation profiles of both SEGA and control samples. principal component analysis (PCA) of the methylation data in SEGA (n=42) and periventricular control tissue (n=8) showing that the major source of variability in CpG methylation is the diagnosis. *x*-axis: the first principal component (PC); *y*-axis: the second PC. Labeling the age per sample (**a**), labeling the mutation per sample (**b**), labeling the gender per sample (**c**) and labeling the batches per sample (**d**). **e.** shows that there is no significant difference in age between control and SEGA samples. (TIF 1459 kb)

Supplementary Figure 3. Robustness of the two SEGA groups in extended cohort of SEGAs. TSNE plot (**a**) and heatmap (**b**) with a total of 92 SEGAs, showing the robustness of the two SEGA groups identified. (TIF 5060 kb)

Supplementary Table 1. GO terms enriched in SEGA compared to control tissue using missMethyl. (XLSX 83 kb)

## Data Availability

The datasets used and/or analysed during the current study are available from the corresponding author on request.

## Abbreviations

TSC: Tuberous sclerosis complex
mTOR: The mechanistic target of the rapamycin complex
SEGA: Subependymal giant cell astrocytomas
SEN: Subependymal nodules
TBC1D7: TBC1 domain family member 7
GAP: GTPase-activating protein
RHEB1: Ras homolog enriched in brain 1
ECM: Extracellular matrix
MAPK: Mitogen-activated protein kinase
DNMTs: DNA methyltransferases
CNS: Central nervous system
450k: Illumina Infinium HumanMethylation450 BeadChip
PCA: Principal component analysis
PVCA: Principal variance component analysis
ROC: Receiver operating characteristic
IGR: Intergenic region
GO: Gene ontology
GFAP: Glial fibrillary acidic protein
MAP2: Microtubule-associated protein
HLA-DP, DQ, DR: Human leukocyte antigen class II
CD3: Cluster of differentiation 3
pS6: Phospho-S6 ribosomal protein
ROI: Regions of interest
LOH: Loss of heterozygosity
