# Supplementary figures and images for: Distinct DNA Methylation Patterns of Subependymal Giant Cell Astrocytomas in Tuberous Sclerosis Complex
Source: Cell Mol Neurobiol. 2021 Oct 28;42(8):2863–92. doi: 10.1007/s10571-021-01157-5 (PMC9560915; doi:10.1007/s10571-021-01157-5)

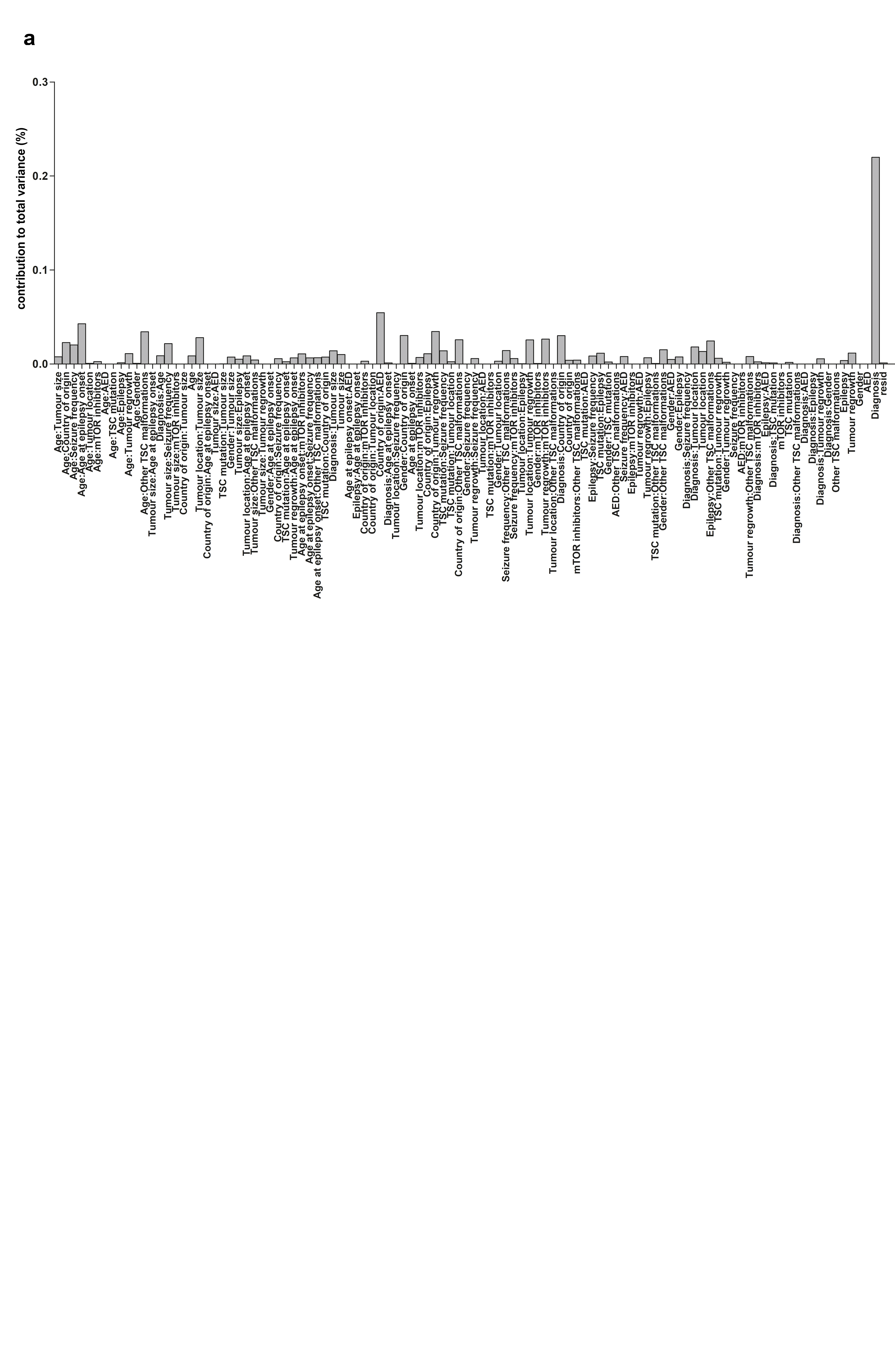

Supplement: Supplementary file 1 — Supplementary Figure 1. Principle variance component analysis (PVCA) of all the methylation profiles of both SEGA and control samples. a. A PVCA was performed to quantify the contribution of various variables to the overall variance between the samples (SEGA n=42 and control tissue n=8). The major contributor to the overall variance was the diagnosis of the samples (SEGA or control). Other clinical data including age at surgery, TSC1/TSC2 mutation status, gender, localization of the SEGA, size of the tumor, epilepsy, age at seizure onset, seizure frequency, drug management at time of surgery (including treatment with mTORC1 inhibitors), tumor recurrence/regrowth and presence of other TSC related malformations as well as their various interactions contributed minimally to the overall variance seen amongst the samples. The residual is all variance that cannot be explained by the known factors. b. A PVCA was performed to quantify the contribution of various variables to the overall variance between the SEGA samples (SEGA n=42). The major contributor to the overall variance of the SEGA samples was the three subgroups (SEGA1, SEGA2a, SEGA2b) found based on the methylation profile, followed by the two subgroups (SEGA1, SEGA2). Other clinical data including age at surgery, TSC1/TSC2 mutation status, gender, localization of the SEGA, size of the tumor, epilepsy, age at seizure onset, seizure frequency, drug management at time of surgery (including treatment with mTORC1 inhibitors), tumor recurrence/regrowth and presence of other TSC related malformations as well as their various interactions contributed minimally to the overall variance seen amongst the samples. The residual is all variance that cannot be explained by the known factors. (TIF 6492 kb) [file 10571_2021_1157_MOESM1_ESM.tif]

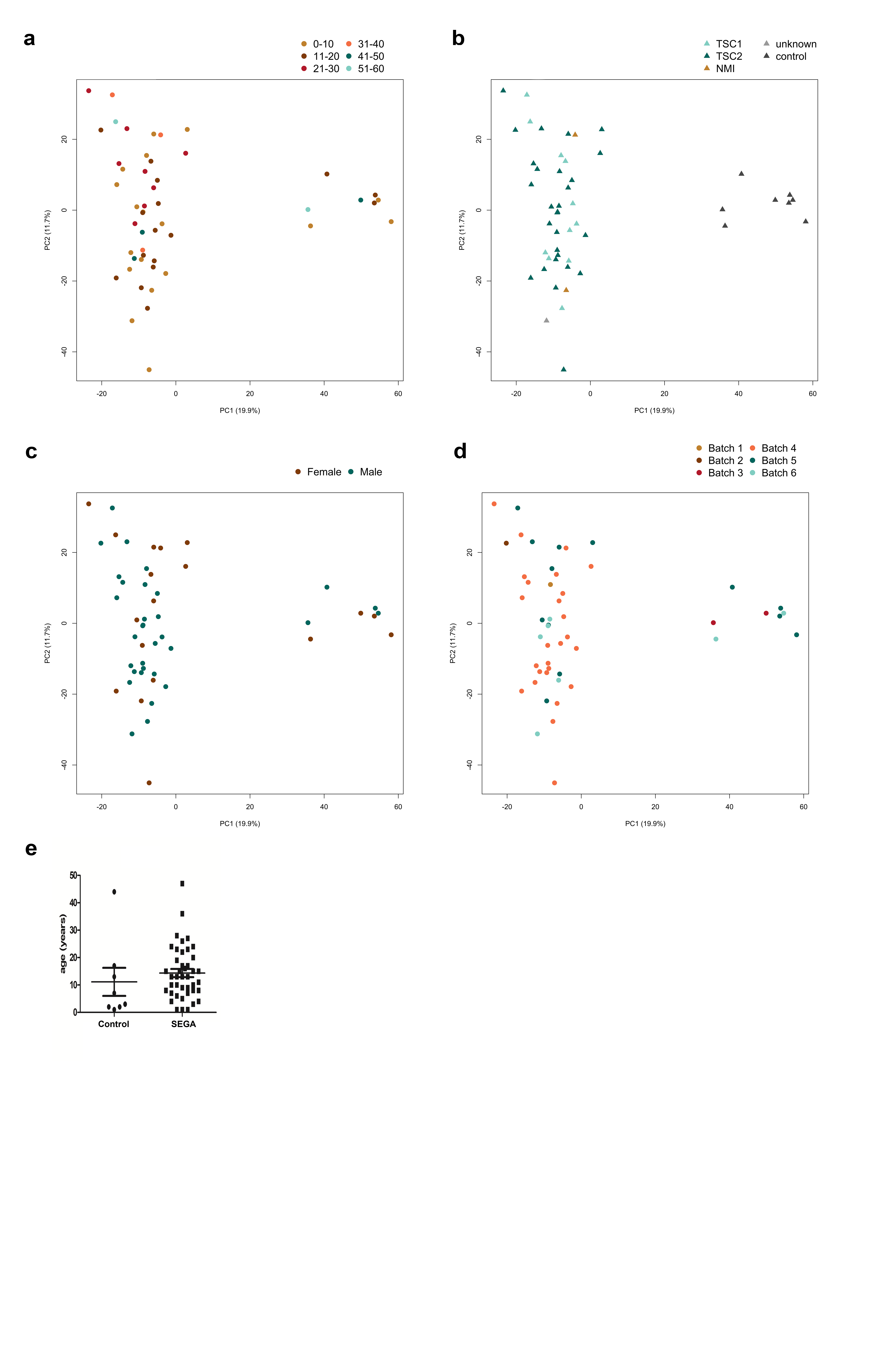

Supplement: Supplementary file 2 — Supplementary Figure 2. Principle component analysis (PCA) of all the methylation profiles of both SEGA and control samples. principal component analysis (PCA) of the methylation data in SEGA (n=42) and periventricular control tissue (n=8) showing that the major source of variability in CpG methylation is the diagnosis. x-axis: the first principal component (PC); y-axis: the second PC. Labeling the age per sample (a), labeling the mutation per sample (b), labeling the gender per sample (c) and labeling the batches per sample (d). e. shows that there is no significant difference in age between control and SEGA samples. (TIF 1459 kb) [file 10571_2021_1157_MOESM2_ESM.tif]

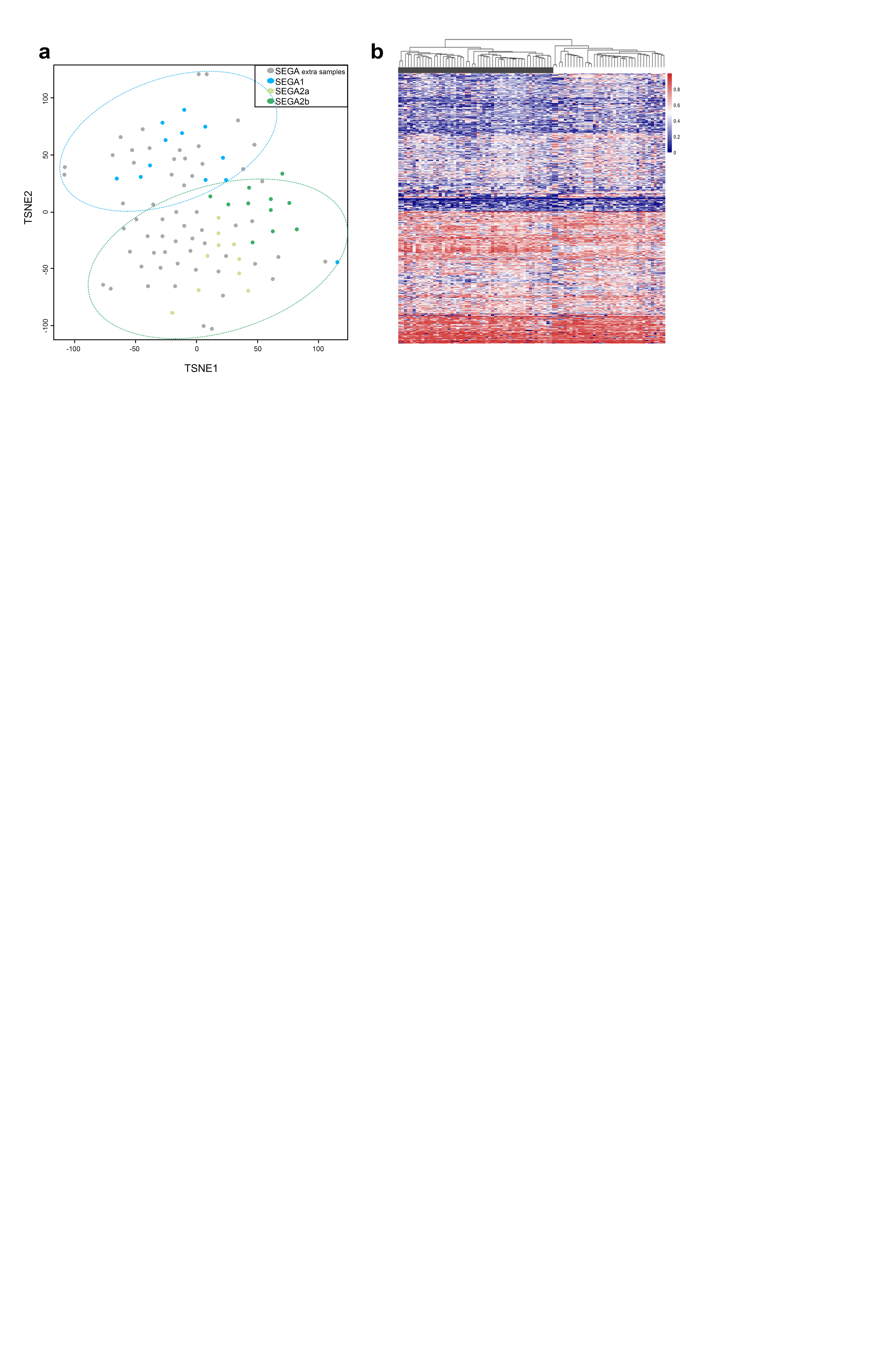

Supplement: Supplementary file 3 — Supplementary Figure 3. Robustness of the two SEGA groups in extended cohort of SEGAs. TSNE plot (a) and heatmap (b) with a total of 92 SEGAs, showing the robustness of the two SEGA groups identified. (TIF 5060 kb) [file 10571_2021_1157_MOESM3_ESM.tif]
